# Livestock immunomodulators as alternatives to antimicrobials – a roadmap for the fight against drug resistance

**DOI:** 10.1186/s12917-026-05619-0

**Published:** 2026-06-16

**Authors:** Daniel S. Ackerman, Alex Morrow, Adrian L. Smith, Gary Entrican

**Affiliations:** 1 Insight Editing London, London, UK; 2 CABI, Nosworthy Way, Wallingford, Oxfordshire OX10 8DE UK; 3 Department of Biology, Medawar Building, University of Oxford, South Parks Road, Oxford, OX1 3SY UK; 4 The Roslin Institute at University of Edinburgh, Easter Bush Campus, Edinburgh, Midlothian EH25 9RG UK

**Keywords:** Adaptive immunity, Alternatives to antimicrobials, Antimicrobial peptides, Antimicrobial resistance, Host defence peptides, Host-pathogen interactions, Immunomodulators, Innate immunity, Phytochemicals, TLR agonists

## Abstract

Antimicrobials are currently an indispensable pillar of the animal production industry, relied upon to treat diseases that could otherwise severely impact animal welfare and productivity. However, overuse of antimicrobials is driving the selection of drug-resistant microbes. Coupled with the rising demands placed on animal production systems by the growing human population, we now face an increasingly urgent need for new strategies to address animal diseases and their impacts on productivity, efficiency, and welfare. The identification and development of alternatives to antimicrobials is therefore a critical and fast-growing field of research. The STAR IDAZ International Research Consortium recently conducted workshops that recruited international experts to develop research roadmaps aiming to stratify, organise, and clarify the ongoing research and outstanding questions that currently define the field of alternatives to antimicrobials. This review will explore the roadmap that deals with immunomodulators, a broad class of technologies that aim to beneficially alter host immunity in ways that promote animal health and growth, mitigate disease, and improve welfare at the human-animal interface. We will discuss the STAR IDAZ immunomodulators roadmap in detail, addressing the foundational biology underlying immunomodulation; the fields of research that link livestock immunity and metabolism with response to infections; and the immunomodulator technologies that are currently under development.

## Background

As the global food production system continues to expand to meet the needs of the growing human population, treatment and prevention of animal disease are essential for ensuring robust animal welfare, reducing the impact of infectious disease transmission (to animals and the consumer), and maintaining high levels of animal product output. The many animal-animal, animal-nature, and animal-human interfaces inherent to farming and food production expose livestock to a great variety of pathogens capable of spreading through the environment, through direct or indirect contact, and/or via secondary vectors like ticks or mosquitoes. Farming systems can also alter the profile of the pathogens that afflict livestock, either by changing transmission opportunities or by pathogen adaptation and evolution to the farm environment [1–5]. The consequences of the resulting infections can range from mild to lethal, and all can potentially result in reduced animal productivity and quality of life. The farm environment often results in changing opportunities for infectious disease transmission between animals, in part due to the need to co-house groups of animals; this leads to increased contact and opportunities for transmission of many infections. The production environment (farm, market, and processing) can also increase the transmission of infection between animal species, particularly when intensification of production is not accompanied by improved biosecurity. An equally pressing concern is zoonosis – diseases transferring from animals to humans (or vice versa), facilitated by necessarily high levels of contact between livestock and food production workers. Zoonosis has been a regular occurrence since prehistory [6], and increasing populations of humans and livestock provide more and more opportunities for pathogens to evolve the genetic adaptations required to jump between species. Approximately 60% of modern human pathogens are thought to be zoonotic, rising to 60–75% in emerging infectious diseases [7, 8].

Reduction of these risks is a critical pillar supporting animal agriculture and public health, and antimicrobials have long been our primary tool for treating and preventing infectious diseases of livestock [9]. This pillar is currently being chipped away by the growing problem of antimicrobial resistance (AMR). Animal disease is not a static target – high genetic mutation rates are a hallmark of invasive animal pathogens, allowing the rapid selection of strains resistant to widely used drugs [10]. These AMR pathogens are not limited to animals and can spread to humans and the wider environment via direct contact, animal products, and environmental contamination [9, 11–14]. Calls to limit AMR by reducing antimicrobial use in livestock have persisted for decades [15–19], prompting governments to regulate their widespread and/or prophylactic use. Antibiotic usage in livestock has since been restricted and regulated in many countries, including major animal producers like the European Union [20, 21], the United States [21, 22], Brazil [23], and China [24]. However, the ever-growing need for higher quantity and efficiency in food production continues to coincide with vast overuse of antimicrobials in livestock – particularly their administration in feed as a preventative and to promote growth [15]. Antimicrobial use in livestock remains partially to completely uncontrolled in many regions including much of Africa, Southeast Asia, and parts of China [25–29]. This trend is expected to continue through the current decade [30, 31], with lower-income countries taking the brunt of the consequences of widespread AMR [11, 32]. Many possibilities are being explored to mitigate this urgent problem and protect animal agriculture, including regulatory avenues – e.g., restrictions on antimicrobial use and enhanced livestock disease surveillance – and biological research, including vaccinations and the discovery of new antimicrobials and drug discovery platforms [17, 32–34]. To support this ongoing research, the STAR IDAZ International Research Consortium (IRC) recently produced a set of research roadmaps for developing Alternatives to Antimicrobials (ATA) [35], covering three particularly promising areas of research: (1) microbiota optimisation, (2) phage technologies, and (3) immunomodulators, the third of which is the topic of this review.

The history of immunomodulation – the alteration of immune responses to treat disease and alleviate symptoms – stretches back for centuries in various forms [36, 37], but its widespread application to veterinary species is a relatively young field [38–40]. This review will focus on non-vaccine immunomodulators, which include a broad range of immunoactive agents such as exogenous immunoglobulins and cytokines, nanoparticles, extracellular vesicles (EVs), host defence peptides (HDPs), antimicrobial peptides (AMPs), proinflammatory microbial products, pattern recognition receptor (PRR) agonists, and gene and cellular therapies (reviewed in [38, 41]). These immunomodulators stimulate innate and/or adaptive immunity to directly and/or indirectly promote pathogen clearance, reducing symptoms and mortality due to infection. Immunomodulators therefore mitigate the risks of AMR by stimulating the natural immunity of the treated animal, reducing reliance on antibiotics.

Development of these immunomodulators – the identification of effective agents, their validation in vitro/in vivo, and their application in the field – is built on our knowledge of host-pathogen interactions, immuno-developmental biology, immune/inflammatory balance, and the regulation and metabolic cost of the immune response. Meanwhile, the costs of bringing an immunomodulator to market are substantial to both companies and veterinary stakeholders. These costs come from a multitude of sources, some of which are specific to the type of immunomodulator (e.g., the costs of recombinant protein or nanoparticle production) while others are more general (e.g., company production capacity, product validation and safety testing, and the costs and complexities involved in obtaining regulatory approval for new drug additives). Additionally, economic incentives are often lacking for veterinary vs. human therapeutics, and the need to test therapeutics in multiple animal species and/or breeds can place additional burden on the production pipeline (reviewed in [42–46]). Effective research requires careful consideration of the host animal, the specific disease(s) being targeted, the state of the animal market, and ongoing trends in epidemiology when balancing these costs against potential benefits. The development of immunomodulators for food animals must also address potential risks to the consumer, requiring that accumulation of the therapeutic in animal products and its safety to humans both be assessed [47, 48]. To date, only four non-vaccine immunomodulators have received regulatory approval for animal agriculture in the United States (reviewed in [38, 49]): Amplimune (approved in 1993), a mycobacterial immunostimulant to reduce the severity of K99^+^ *Escherichia coli*-associated colibacillosis in cattle [50]; Zylexis (approved in 2005, later discontinued), an inactivated poxvirus to promote immune response to equine herpesvirus [51–53]; Zelnate (approved in 2015), an immunogenic DNA liposome complex to reduce mortality due to *Mannheimia haemolytica*-associated bovine respiratory disease [54, 55]; and Imrestor (approved in 2016, no longer marketed), a modified recombinant bovine cytokine (rbG-CSF; granulocyte colony-stimulating factor) to restore immune activity and reduce mastitis in dairy cattle following parturition [56].

**Fig. 1:**
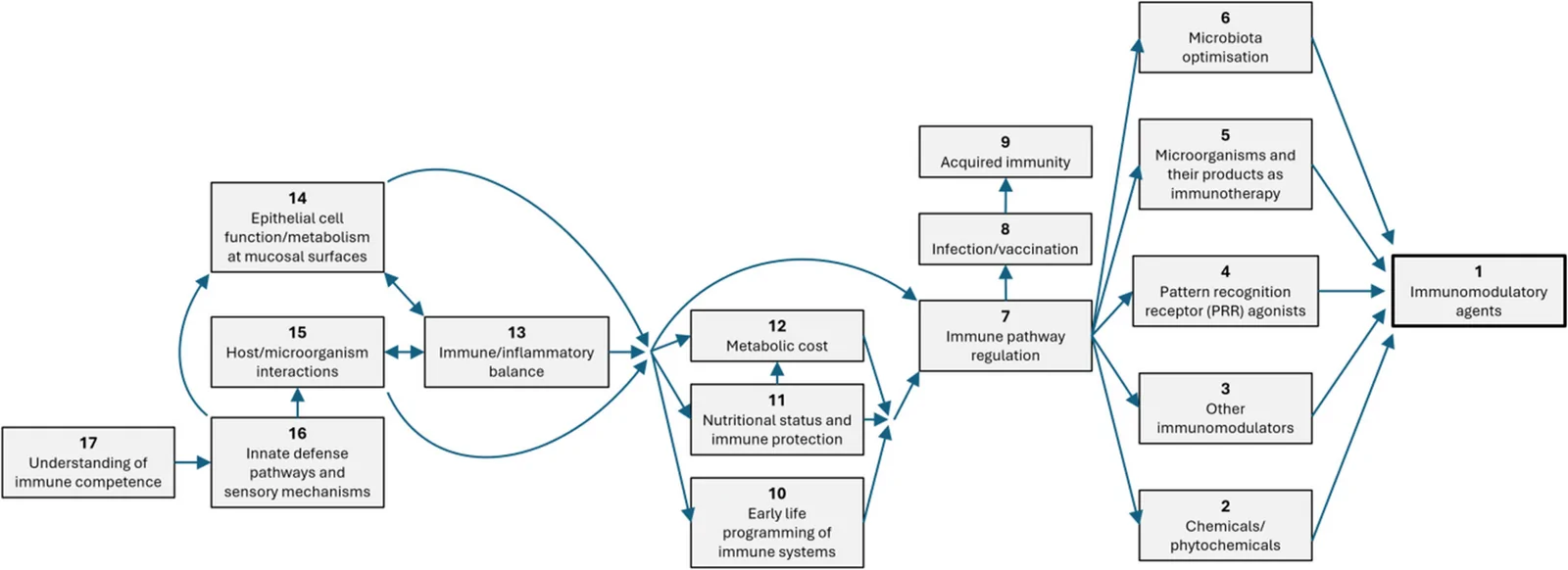
The STAR IDAZ antimicrobial resistance and alternatives to antimicrobials roadmap for immunomodulators. Each numbered node represents a field of research and/or development of particular importance for livestock immunomodulation. On the right, the roadmap shows the various technologies that are currently being developed as immunomodulatory agents to promote immune activity and effective disease response in animals. This development is built on and supported by (indicated by arrows) ongoing research into the fundamentals of livestock immunocompetence, innate immunity and the host-pathogen interface, and the host factors that impact immunity

The paucity of available non-vaccine veterinary immunotherapies evidences the difficulty and cost of developing effective agents and facilitating their widespread use in veterinary medicine [38]. Nevertheless, the collision of increasing AMR prevalence with the expanding need for output and efficiency in the livestock industry is creating new opportunities for immunotherapy. The STAR IDAZ ATA immunomodulators roadmap (Fig. 1) was constructed to help guide this growing field of research by collating the most important research fields involved in livestock immunomodulator development and highlighting important challenges and knowledge gaps that remain to be filled. This review will be based on that roadmap and will describe and explore the research fields included therein, including the immunological background, ongoing research avenues, and classes of immunotherapy agents that shape the current field of livestock immunomodulation.

## Main text

### Understanding immunocompetence – node 17

Developing effective immunomodulation strategies relies on a comprehensive understanding of the physiological mechanisms of disease resistance. Immunocompetence – the ability to mount an immune response against infection – is a complex and multifaceted property that is closely interlinked with both internal (e.g., genetics, age, physiology, past exposure to pathogens, and stress levels) and external (e.g., environmental conditions) phenomena [57, 58]. Efforts to reduce disease severity by modulating immunity must therefore grapple with the idiosyncratic nature of the immune response, which can vary among animals (hosts), pathogens, and environments [59]. Recent advances in genomics, gene editing, and cellular biology have facilitated increasingly detailed efforts to understand the evolved differences in immunity among species through the field of comparative immunology [60]. Farm animals have served as important models for the development of these technologies and for their comparative applications [61, 62].

This inter-animal variability in immune response (evident in evolutionary changes across species and in genetically or environmentally induced differences between strains or breeds of a single species) has also spurred the use of selective breeding as a long-term form of livestock immunomodulation. Selection for positive traits has long been an integral part of animal farming, typically applied to traits directly associated with profitability (such as milk yield in dairy cows or feed conversion rates in chickens); more recently, our increasing understanding of immunogenetics has led to the same selection strategies being applied to livestock and poultry to increase their resistance to infection or resilience to disease [63, 64]. Numerous groups have developed quantitative measures of immune resilience, combining various physiological variables – e.g., blood leukocyte counts, antibody production, and the strength of humoral and cell-mediated immunity in response to vaccination or infection (reviewed in [65]) – to be used as proxies for overall immunocompetence in selective breeding [64, 66–71]. These quantitative measures of immunocompetence are particularly important given that selective breeding for growth can introduce unintended changes that negatively impact immune resilience (and vice versa), or breeding for resistance to one disease may negatively affect resistance to another [72–74]. This requires that a delicate balance be struck between immune response and more easily observable growth-/productivity-related variables. In layer chickens, for instance, breeding for increased response to inactivated Newcastle disease virus (NDV) was linked to reduced body weight and residual feed intake [75], while breeding of broilers for increased feed efficiency adversely impacted innate immunity [76]. The mechanisms that underlie these outcomes remain undefined, but they may be due to genetic linkage between production- and immune-related genes or broader physiological relationships. Given the potential impact of genetic (e.g., polymorphisms in innate immune genes such as pattern recognition receptors) and environmental factors on the outcomes of immunomodulators, it will be useful to perform studies taking into account both of these factors. Indeed, inspiration and approaches can be drawn from the fields of ecoimmunology (the study of wild animals in diverse environments) and evolutionary immunology [77–79]. Immunogenetics is proving very powerful in this arena and could be more intensely applied to livestock species, particularly where the genetic variation is combined with functional analyses of variants. Genetic variation in immune genes is being supported by the development of large datasets of full genome sequences for some livestock species (e.g [80]), , and similar resources could be developed for other species. Predictable outcomes from interventions will be supported by understanding the genetic and functional diversity in immune genes both within and across breeds or species whilst integrating this information with outcomes in different environments.

While efficiency in growth and output continues to grow through selective breeding, these trade-offs between productivity and immunocompetence emphasise the increasing need for research into the host- and pathogen-specific mechanisms of immunity and its associated metabolic costs. Meanwhile, AMR is placing new and urgent pressures on the entire system by threatening the effectiveness of indispensable antimicrobials that have been relied on to support productivity and supplement host immunity. The STAR IDAZ immunomodulators roadmap (Fig. 1) is intended to provide a blueprint for navigating this complex research area and applying new findings to the development of immunomodulators that can sidestep the unpredictability of selective breeding by directly boosting immunocompetence. The following sections will discuss, through the lens of this roadmap, the research fields that comprise livestock immunomodulation.

### Innate immunity and the host-pathogen interface – nodes 14–16

While the direct role of the host immune system in veterinary infections has been extensively studied, recent technological developments have allowed us to begin distinguishing and studying more granular host- and pathogen-specific properties including the multifunctionality and redundancy of the host immune system, genetic and epigenetic control of immune responses, and pathogen evolution during infection (reviewed in [59]). The explosion of next-generation parallel sequencing technologies over the past two decades has also benefitted our understanding of the myriad microbes and viruses to which farm animals are exposed. Bovine respiratory disease (BRD), for instance, is a major economic burden on cattle farming and has an infamously complex aetiology, involving numerous bacterial and viral pathogens interacting with animal and environmental conditions and farm management practises (reviewed in [81]). Second- and third-generation sequencing technologies offer new ways to identify co-infecting pathogens of the BRD complex and to explore the host immune response via metagenomics [82, 83], RNA-Seq [84], microbial 16 S ribosomal RNA and shotgun sequencing approaches [85], etc. (reviewed in [86]). In a broader context, the ever-lowering cost and increasing diversity (e.g., short- versus long-read technologies) of parallel sequencing approaches and the development of new single-cell and shotgun technologies provide new opportunities for greater dissection of the disease process and the role of different immune components in developing resistance or resilience. Importantly, as with many developing diagnostic technologies, the standardisation, cost-effectiveness, and field applicability of these methods will be vital for promoting widespread use.

### Rapid response: early cell-pathogen interactions and the mucosal barrier

As these new technologies facilitate increasingly comprehensive studies of animal immune systems and the infectious agents to which they are exposed, immunomodulation finds its niche at the interface between host and pathogen, where the many interactions that determine the course of infection and pathogen clearance provide specific points where modulators could potentially influence disease progression. Many relevant studies have focused on the Toll-like receptors (TLRs), as this small but evolutionarily ancient family of cell-surface PRRs is a major player in the earliest stages of immune activation [87]. Our knowledge of TLRs in farm animals has expanded rapidly over the past two decades [88–90], and TLR agonists are now being explored as immunomodulatory agents for a host of economically relevant diseases of farm animals including bovine mastitis, porcine respiratory syndrome, and avian Newcastle disease (reviewed in [89, 91, 92]). Specific examples of TLR immunomodulation will be discussed later (see “Modulating PRR activity” below). It is important to note that studies of veterinary TLR agonists can often display inadequate reporting of statistics, potential for bias, and other methodological limitations [89]; addressing these shortcomings, which are particularly troublesome given the difficulty of controlling field research, will be an important step for future research.

Many host-pathogen interactions, including TLR activation, occur at the sites of infection or exposure to immunomodulators including at the mucosal barriers (e.g., in the respiratory system and intestines), where inhaled or ingested pathogens first encounter innate immune cells of the host animal. The functions, properties, and cell populations of the mucosal immune system in relevant animal species have been comprehensively reviewed elsewhere [73, 93–98]; here, we will address some specific studies that target research gaps and challenges in the application of immunomodulators to mucosal immunity. Many of these have investigated animal diet as an avenue for passively modulating innate immune responses. In cattle, for instance, whole transcriptomic sequencing identified significantly lower activation of jejunal mucosal immunity in calves experiencing restricted milk access [99], underscoring the importance of early-life diet for establishing proper crosstalk between metabolism and immunity (reviewed in [100, 101]). Bovine colostrum has also been found to have beneficial immunomodulatory effects in vitro, inducing anti-inflammatory Th2-shifted cytokine changes and promoting intestinal barrier integrity in response to *Salmonella typhimurium* infection [102]. In aquaculture, meanwhile, the polysaccharide β-glucan has been studied as a feed additive with bioactive immunostimulant properties [103]. Dietary supplementation has also been explored for enhancing early immune responses to specific pathogens, including via bioactive compounds like inulin, which augmented porcine mucosal barrier integrity and immunity in response to infection with the whipworm *Trichuris suis* [104]; natural products like *Glycyrrhiza* polysaccharides, which protected against intestinal injury in a mouse model of pseudorabies virus infection [105]; and pro/prebiotics that alter the gut microbiome to promote immunity. This last field in particular in a huge and growing area of active research that cannot be covered in sufficient detail here, but a great number of extensive reviews have recently been published to summarize the ongoing development of microbiome-adjusting immunomodulators for animal use (e.g [106–112]), . As dietary strategies for immunomodulation continue to develop, in vivo standardisation and validation studies will also grow in importance, and any diet changes intended to promote innate immunity will need to be balanced against their effects on productivity variables (weight gain, body composition, etc.) under field conditions.

Mucosal immunomodulation may also provide opportunities to ameliorate infection risk during periods of stress; in Holstein calves, for instance, heat stress disrupted the intestinal mucosal barrier, but concurrent treatment with dexamethasone rescued this effect [113]. In fish, the many unnatural stressors involved in high-intensity aquaculture have been shown to have deleterious effects on mucosal barrier integrity and immunity [114–116]; a variety of dietary supplements and additives have been investigated for counteracting these effects, but we broadly lack species-specific understanding of dietary immunomodulation in fish (reviewed in [117]).

### Epithelial cells in the early immune response

Epithelial cells form the bulk of these initial barriers against pathogens, with specialised populations producing various “barrier molecules” [118] with direct and indirect antimicrobial properties; these include the mucins that impede microbial attachment and the broad class of AMPs that directly interact with invading pathogens. Their critical role in the innate immune response makes epithelial cells particularly important for understanding early host-pathogen interactions and immune stimulation/regulation and for identifying potential immunomodulators. Host-derived AMPs in particular constitute an especially active area of immunomodulation research and will be discussed in greater detail below (see “Altering host-pathogen interactions”).

Ayalew et al. recently reviewed research on the *in ovo* delivery of immunomodulatory supplements to chicks [119]. Administration of various pre/probiotics and organic acids has been associated with improved gastrointestinal tract epithelial barrier integrity and improved post-hatching viability and growth; however, many discrepancies exist among published data, and the mechanistic studies necessary to evaluate these differences are currently lacking (reviewed in [119]). Interactions between immunobiotics and intestinal epithelial cells are being studied for antimicrobial and antiviral effects in cattle, especially during the birth-to-weaning period when calves are considered highly vulnerable to infection (and are thus likely to be treated with prophylactic/metaphylactic antibiotics) (reviewed in [120]). A particularly important point is that the neonatal immune system is quite different from the fully developed adult immune system. This is particularly relevant where the entire production process is focused on young animals – e.g., broiler chickens reared for meat production, which are harvested before developing full immune maturity.

A new generation of in vitro experimental models that inform on in vivo epithelial-immune dynamics is emerging. For example, Karaffová et al. developed an ex vivo chicken ileal explant model for investigating mucosal immunity. Initial findings with this model indicated that the probiotic *Limosilactobacillus reuteri* B1/1 can modulate gene expression patterns in the intestinal mucosa to prevent infection with *Salmonella enterica* serovar Enteritidis [121]. Meanwhile, new bovine intestinal [122] and mammary [123] epithelial cell lines may facilitate immunological studies of mucosal immunomodulation against enteric diseases and mastitis.

 In vitro studies are especially important in immunomodulation research, as they can isolate the response of epithelial barrier cells to treatment with modulators that can have both positive and negative effects on their metabolism and proliferation. One example comes from a recent study of proinflammatory cytokines in bovine small intestinal organoids, where 24-hour treatment with TNF-α or IFN-γ significantly reduced barrier integrity and epithelial cell proliferation [124]. This is not a universal effect of proinflammatory cytokines as, notably, IL-18 treatment did not affect these properties [124], indicating the complex, multi-layered nature of the biological response to inflammatory stimuli even under isolated conditions. Similar studies have been conducted in cultures of epithelial cells originating from the bovine mammary gland [123], oviduct [125], and rumen [126], and the avian intestine [127, 128].

### Host factors impacting immunity – nodes 10–13

These in vitro studies illustrate the complexity of the host immune response from its earliest stages. As infection progresses, the crosstalk between inflammation and other systems of homeostasis adds yet more layers to the complicated network of signalling molecules and response pathways that are the targets of interest for immunomodulation. Here, we will address two physiological systems in particular that are heavily interlinked with immunity – metabolism and the endocrine system – while noting that the immune response is ultimately a whole-body reaction that necessitates a big-picture view when investigating potential immunomodulators.

### Nutritional and metabolic requirements

As discussed above, the propensity of the immune response to both help and hinder barrier cell populations is an important consideration when identifying or testing modulators of that response. Immune system homeostasis incurs a metabolic cost that can negatively impact animal fitness. Furthermore, “optimal immune function may not necessarily be maximal immune function” [72]; the damage done by an overactive immune system can outweigh the benefits of stronger responses to infection [72, 129, 130]. As with all nutritional supplementation strategies, moderation is key for dietary immunomodulation; some nutrients (e.g., vitamin D and omega-3 fatty acids) may have anti-inflammatory benefits at levels above current recommendations, while others may “over-modulate” (reviewed in [131]). The multifunctional nature of immunometabolism makes the potential effects of dietary supplementation difficult to predict and sensitive to differences in environment, animal breed and age, etc [132, 133].

Targeting specific stressors and/or known periods of high risk for animals is one strategy for mitigating this complexity. As noted above, heat stress is known to weaken the intestinal barrier in cattle [113]. This damage occurs through both immune and metabolic responses to heat, including increased cytokine production and reduced fatty acid and amino acid metabolism, leading to productivity loss [134–136]. A recent study from Ban et al. addressed heat stress with dietary sucrose supplementation, which partially restored pro- and anti-inflammatory immune cell balance and ruminal metabolism [134]. Much research has also been focused on the peripartal period in cattle, when the nutritional and behavioural adaptations that characterise late prepartum through early postpartum are associated with significant metabolic and immune imbalances that threaten the health of the animal [137–142]. Dietary supplementation with medium-chain fatty acids was reported to regulate cytokine production and decrease inflammatory marker serum levels; notably, this also resulted in higher milk fat percentage but lower milk production in early lactation, illustrating another trade-off between immunometabolic and strict production variables [143]. Modulators of one-carbon metabolism, a key biochemical process that generates single-carbon metabolites for various anabolic pathways, have also been investigated for their ability to alleviate peripartal risk of metabolic and immune disorders. Supplementation with methyl donors (particularly methionine and choline) appears to have positive effects on liver function and oxidative stress, though the precise mechanisms linking these effects to the wide array of immunometabolic, epigenetic, and endocrine changes associated with methyl donor supplementation remain unclear [132].

### The endocrine system and the immune response

The endocrine system is a direct link between environmental stressors and inflammation, making it a key factor in understanding the animal immune system and developing new immunomodulators. Physiological stress in its many forms is a continuous and inevitable factor in animal farming, and the short- and long-term physiological responses of animals to stressors – including temperature, infection, crowding, inability to perform natural behaviours, and many other variables – all impact the immunological state of these animals. Many recent studies have explored the interconnections between the endocrine and immune systems in economically significant animals including cattle [144–146], pigs [147], horses [148], poultry [147, 149], and fish [130]. Across these species, broad patterns are visible: the effects of stress are generally immunosuppressive, with activation of the hypothalamic-pituitary-adrenal axis leading to the release of corticosteroids that regulate general metabolism and also impact immune metabolism/function, resulting in effects such as lymphopenia at varying levels [150]. These effects can be observed at the animal and cellular levels – in neonatal pigs, for instance, a single 4-hour period of social isolation was associated with increased sensitivity to lipopolysaccharide (LPS) exposure and reduced cortisol sensitivity in peripheral blood mononuclear cells (PBMCs) [151].

The most direct means of reducing stress-associated immunosuppression is to reduce the number and severity of stressors encountered by animals. These efforts go hand-in-hand with improving animal quality-of-life. This field of research is beyond the scope of this review, but many extensive reviews are available covering decades of studies (e.g [144, 152–155]), . The immunomodulatory perspective focuses less on preventing stress and more on managing the resulting immunological impacts.

Endocrine responses to stress overlap intricately with immunometabolism, and dietary supplementation is a widely studied route of beneficial modulation here as well. Supplementation of colostrum with omega-3 fatty acids was reported to reduce oxidative stress and promote immune development in newborn calves [156, 157], and various dietary supplements (e.g., vitamins A and E, selenium, and methionine) have been studied as immune-bolstering supplements to offset the negative consequences of heat stress in cattle (reviewed in [146]). In lactating cows, the immunomodulatory supplement OmniGen-AF – a proprietary mix of trace minerals, vitamins, and microbial components – has been reported to reduce serum cortisol concentrations [158], though conflicting results on the contributing effects of heat stress show the difficulty of isolating specific immunomodulatory effects under field conditions [159]. In pigs, positive modulation of stress-associated immunosuppression has been reported via dietary supplementation with L-arginine [160] and other amino acids (reviewed in [147]), fatty acids [161], vitamins [162, 163], and trace elements [164].

Also important to consider are the unintended endocrine/immunomodulatory effects of environmental contaminants, which have been studied extensively in fish. Endocrine-disrupting chemicals (EDCs) – stemming from industrial waste, pharmaceuticals, cosmetics, pesticides, and many other sources – are widespread contaminants of freshwater ecosystems [165, 166], and the hormonal imbalances caused by these bioactive chemicals can significantly impact immunity as well. The accumulation of EDCs in fish can have unpredictable consequences for the health of the animals; xenoestrogen exposure, for instance, has been associated with disrupted thymus development in several fish species [167–169] while unexpectedly reducing mortality in largemouth bass (*Micropterus salmoides*) infected with the pathogenic Gram-negative bacteria *Edwardsiella piscicida* [170]. Meanwhile, the oestrogen receptor-disrupting organotin compound tributyltin has been linked to a constellation of negative immunological consequences in fish, including cytokine alterations, thymic atrophy, and unbalancing of lymphocyte populations (reviewed in [171]).

### Modifying the immune system to fight disease and dysfunction – nodes 7–9

The studies discussed above are a small slice of the rapidly expanding field of research into livestock immunomodulation, illustrating the substantial strides that have been made and the many questions that remain to be answered regarding the mechanisms of immunity and the reagents capable of immunomodulation in specific conditions and animals. In this section, we will briefly address two separate research avenues that are closely linked to immunomodulation – vaccine development and innate immunity training – before closing by discussing specific classes of reagent that are currently the subject of research as livestock immunomodulatory agents.

Nodes 8 and 9 of the STAR IDAZ immunomodulators roadmap (Fig. 1) deal with acquired immunity and vaccination, a vast research field in which steady progress is being made to expand our repertoire of available vaccines, improve the safety and efficacy profiles of existing vaccine candidates, and improve cost-benefit ratios to promote vaccine uptake. This research is itself the subject of a separate STAR IDAZ roadmap [172] and has been extensively reviewed in the specific context of combating AMR [173–182].

At the interface between the acquired immunity stimulated by vaccines and the innate immunity promoted by immunomodulators is the field of innate immunity training. Over the past three decades, a growing list of studies have demonstrated the existence of lymphocyte-independent immunological “memory” or “innate immune memory” [183, 184] (more appropriately called “trained immunity” [185]) wherein pathogen exposure causes epigenetic responses that can be retained in progenitor cells or long-lived tissue-resident cells. These adaptations allow stronger (or different) innate responses to subsequent infection and may be heritable, boosting immunity in offspring as well (reviewed in [185–187]).

As findings in humans and plants begin to be translated to economically relevant animal species, trained immunity has demonstrated potential as an avenue for reducing antimicrobial usage by improving the early immune response to animal pathogens. In layer hens, for instance, Verwoolde et al. conducted in vitro training of isolated monocytes with yeast-derived β-glucan, which boosted the transcriptional response to LPS rechallenge, increased surface expression of the activation marker CD40, and enhanced nitric oxide (NO) production [188]. A similar study conducted in bovine monocytes reported that β-glucan trainability required the presence of non-classical (CD14^−^CD16^++^) monocytes and M1-polarising conditions [189]. Innate training with β-glucan has also been tested in vivo, where it was linked to a 20% increase in survival rate in chickens challenged with *Salmonella enterica* serovar *Typhimurium* [190].

In cattle, the Bacillus Calmette-Guérin (BCG) live attenuated tuberculosis vaccine is known to also induce non-specific protection against other diseases (reviewed in [191, 192]) via trained innate immunity. The McGill lab demonstrated that aerosol BCG vaccination of young calves induced an enhanced cytokine response to TLR agonists, with this training lasting for at least three months [193]. More recently, the same group examined γδ T cells, an unconventional subpopulation that possess both innate and adaptive immune properties and are present in relatively high proportions in cattle; subcutaneous administration of the BCG vaccine altered epigenetic markers in bovine γδ T cells, increasing the accessibility of numerous immune-related gene promoters, and increased cytokine production in response to LPS stimulation [194].

As these findings illustrate – alongside other in vivo studies in goats, pigs, and fish (comprehensively reviewed in [185]) – the burgeoning field of trained immunity is continuing to produce new avenues for non-traditional immunomodulation that, in the case of the BCG vaccine, links adaptive and innate immune protection together. This field especially relies on new sequencing technologies that allow us to quickly identify changes in the epigenetic landscape of cells – e.g., DNA methylation patterns, chromatin accessibility, and histone modifications – that allow innate immune cells to respond more strongly to repeated pathogen exposures, potentially reducing the impact of disease. In most cases (particularly in animals), the precise mechanisms underlying these changes remain unclear, and trained immunity is therefore an important and promising target for in-depth studies of livestock immunomodulation.

### Pathways for immunomodulation – nodes 1–6

**Fig. 2:**
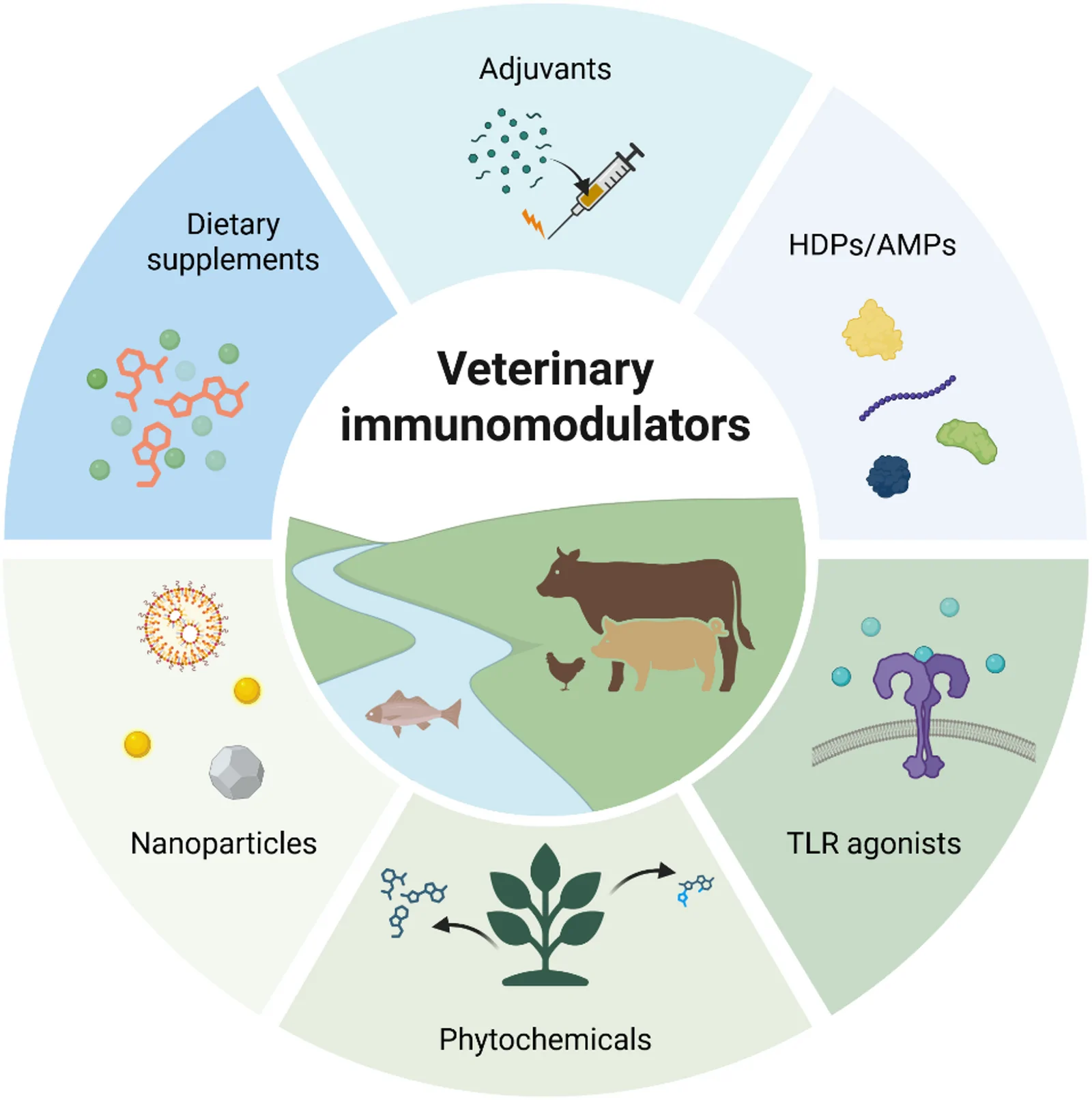
Overview of non-vaccine livestock immunomodulators. Graphical schema illustrates several common classes of non-vaccine immunomodulators: dietary supplements (e.g., vitamins, trace minerals, and bioactives), added to animal feed to balance nutritional deficits, promote proper development, and support immunometabolism; cytokines, which modulate host immune activity and inflammation; HDPs/AMPs, which exert direct and indirect antimicrobial effects to supplement host immunity against pathogens; TLR agonists, which activate innate immune signalling pathways to promote immune response; phytochemicals, plant-derived bioactives with varying pro- and anti-inflammatory effects; and nanoparticles (e.g., metal oxides, gold nanoparticles, etc.), a developing set of technologies with a broad range of potential immunomodulatory effects. AMP, antimicrobial peptide; HDP, host defence peptide; TLR, Toll-like receptor. Created in BioRender. Ackerman, D. (2026) https://BioRender.com/k6lhlno

As the theoretical basis for our understanding of livestock immunocompetence, pathogen response, and immunometabolism continues to grow, these discoveries are being continuously translated into potential immunomodulators through in vitro and in vivo studies. While no single review can adequately describe the entire field of veterinary immunomodulators, we hope that the recent examples discussed below will illustrate some particularly active areas of research and highlight specific developing technologies that fall within the STAR IDAZ immunomodulators roadmap. A high-level graphical overview of non-vaccine immunomodulator technologies is shown in Fig. 2.

### Altering host-pathogen interactions via HDPs and AMPs

The majority of our knowledge on the immunomodulatory activity of HDPs comes from studies of a handful of human HDPs (e.g., β-defensins and the cathelicidin LL-37) [195]. Only more recently have these findings begun to be translated to animals with the goal of reducing antimicrobial usage.

The defensins are a large and widely studied superfamily of cationic HDPs with varying antimicrobial, antiviral, and immunomodulatory functions. The avian β-defensin 8, for instance, was found to upregulate proinflammatory cytokines and chemokines and activate the MAPK signalling pathway in a chicken macrophage cell line [196]. In pigs, β-defensin 129 played an anti-inflammatory role in response to bacterial infection of intestinal epithelial cells, reducing LPS-induced apoptosis and downregulating proinflammatory cytokines [197]. A recent study of porcine β-defensin 5 reported a more subtle immunomodulatory activity in an intestinal epithelial cell line infected with *Brachyspira hyodysenteriae*, involving downregulation of IL-17 and TLR signalling pathways and upregulation of protein metabolism [198].

Many β-defensins have also been identified and are under study for immunomodulatory activity in fish; in the East Asian ayu sweetfish (*Plecoglossus altivelis*), for example, supplementation with synthetic PaBD, a β-defensin homolog, increased survival rate against infection with *Vibrio anguillarum* via modulatory activities in monocytes and macrophages [199]. Other immunomodulating HDPs under study in fish include the piscidins, which are thought to be widely distributed in teleost fish and possess anti-bacterial and -protozoal functionality [200].

The cathelicidin family contains HDPs that are short and amphipathic, like the piscidins, but they are more evolutionarily widespread; cathelicidins have been identified in mammals, avians, and fish (reviewed in [201, 202]), where they stimulate inflammation and play direct antimicrobial roles. Of the four distinct cathelicidins expressed in chickens, CATH-2 has received the most study [203]. Its reported immunomodulatory functions include upregulation of antigen presentation markers on chicken monocytes; LPS neutralisation and reduction of LPS-induced NO production; and promotion of IL-1α and − 1β secretion by neutrophils via activation of the NLRP3 inflammasome [204, 205]. The less-studied CATH-B1 protein has only been confirmed in the bursa of Fabricius [206], a lymphoid hematopoietic organ responsible for the diversification of B cells in birds, but RNA analysis suggests more widespread expression [207]. This HDP was linked to several immunomodulatory activities in primary macrophages exposed to avian pathogenic *E. coli*, with anti-inflammatory effects on cytokine production and TLR4 activation but a more proinflammatory effect on macrophage activation and NO production [203].

Dietary supplementation with LLv, a derivative of the single human cathelicidin LL-37, was also found to promote immune function in broiler chickens [208], though the practical consequences of the reported changes in cytokine expression and antibody secretion have not yet been demonstrated. Meanwhile, the Mátis group has studied the immunomodulatory effects of numerous chicken HDPs in vitro and ex vivo: recently, cecropin A exhibited mixed pro- and anti-inflammatory activities in cultured primary chicken hepatocytes [209] and promoted epithelial barrier integrity in an ex vivo ileal culture [210], where the HDP Pap12-6 was also reported to play concentration-dependent cytokine-modulating and barrier homeostatic roles [211]. It is important to note here that the impacts of cytokine modulation may differ among animal species; the processes by which different cytokines promote Th1/Th2 cell responses in cattle, for instance, are reportedly more complex than those previously observed in mouse models (reviewed in [73]).

Interestingly, chicken CATH-B1 displayed the most significant immunomodulatory effects in a recent study comparing the effects of chicken, pig, and mouse cathelicidins on pseudorabies virus infection in the PK-15 porcine kidney cell line, exhibiting direct antiviral activity and inducing upregulation of IFN-β expression via TLR4 and JNK signalling pathway activation [212]. Finally, Ouyang et al. tested three cathelicidins previously isolated from aquatic species – CATHPb1, Cm-CATH2, and Hc-CATH – in largemouth bass, where they reported antimicrobial properties against relevant bacteria and general proinflammatory activity via TNF-α and IL-1β upregulation [213].

In cattle, many HDPs have been identified and characterised over the past three decades (reviewed in [214]), but studies have rarely progressed to more in-depth analyses of immunomodulatory activity. Bosso et al. recently reported their identification of KNR50, a cationic AMP “hidden” as a cryptic sequence in the C-terminus of the bovine milk protein casein αS2; this peptide exhibited a wide array of immunomodulatory and membrane-targeting antimicrobial effects in vitro and in *Caenorhabditis elegans*, though these activities were not tested in bovine cells [215]. Meanwhile, Saubi et al. conducted a comprehensive evaluation of five bovine HDPs – the β-defensins BNBD1 and BNBD3, lingual antimicrobial peptide, and the cathelicidins Bac5 and BMAP27 – in bovine PBMCs and turbinate epithelial cells, reporting a range of immunomodulatory activities (broadly proinflammatory for BNBD3 and LAP and anti-inflammatory for BMAP27) in response to LPS stimulation [195].

Also important is identifying the factors secreted by infectious pathogens to mediate their interactions with host immune cells. These factors may be promising targets for future immunomodulators – one example is a T63A mutant of the TLR2 activator ESAT-6 secreted by *Mycobacterium bovis*, which was linked to innate immune response in an in vitro co-culture of bovine antigen-presenting cells and lymphocytes [216] – or, in other cases, the microbial products themselves may be useful as immunomodulators. For instance, the *Mycobacterium bovis* multiprotein complex P22PI [217], which includes ESAT-6, was found to bolster immunity in foetal bovine lung cultures at similar levels to whole heat-inactivated bacteria [218], and stimulation with the bacteria-derived TLR1/2 ligand Pam3CSK4 modulated milk phagocytes in dromedary camels [219]. Many other TLR agonists (e.g., unmethylated CpG, double- or single-stranded RNA, LPS, etc.) have been employed as immunomodulators delivered either by injection into tissues or mucosally. These are used often in the context of vaccine adjuvants but also as vaccine-independent immunomodulators (see below).

### Modulating PRR activity

The central role of PRRs such as TLRs in the innate immune response to pathogens makes them an attractive target for developing alternatives to antimicrobials. Beyond their ability to act as potent adjuvants to veterinary vaccines – itself a large and active field of research (reviewed in [220, 221]) – TLR agonists are studied for their immunomodulatory capabilities, enhancing the immune response to specific pathogens. One of the most widely studied PRR immunomodulators is CpG oligodeoxynucleotides (CpG ODN), an agonist for TLR9 in mammals and TLR21 in birds [89]. Administered *in ovo* together with LPS, CpG ODN treatment was found to significantly reduce tumour incidence in chickens infected with Marek’s disease virus. This was tentatively attributed to changes in IL-1β and IL-18 expression, although the mechanism of action is not fully understood [222]. Chen et al. similarly reported that CpG ODN enhanced the antibacterial immune response of humpback grouper fish (*Cromileptes altivelis*) in vitro and in vivo via modulation of *TLR9* and cytokine expression levels [223]. Meanwhile, a combination *in ovo* administration of CpG ODN and TLR2 and TLR4 ligands (Pam3CSK4 and LPS, respectively) reduced avian infectious bronchitis virus replication, with a comparable pattern of cytokine-modulatory effects [224]. The synthetic single-stranded RNA analogue and TLR7 agonist resiquimod also displayed antiviral potential in the MQ-NCSU avian macrophage cell line [225] and in embryonated eggs, where it substantially reduced later NDV replication and promoted proinflammatory cytokine transcription [226].

Another TLR7 agonist, SZU101, has been studied in vivo as an antiviral against porcine reproductive and respiratory syndrome virus (PRRSV), alleviating fever and pulmonary symptoms in PRRSV-challenged piglets and reducing serum viral load; these effects were attributed to enhanced proliferation of anti-PRRSV lymphocytes and increased IFN-β secretion [227]. A dual TLR7/8 agonist (R484) alongside CpG ODN and Pam3CSK4 has also shown potential as an intradermal adjuvant for inactivated PRRSV vaccine [228]. Recently, Franzoni et al. tested the TLR2 agonist Mag-Pam2Cys_P48, a synthetic lipopeptide like Pam3CSK4, as a porcine macrophage immunomodulator against infection with African swine fever virus (ASFV), a highly economically relevant pathogen that was recently detected in wild boar in Spain [229]. Unfortunately, this agonist showed no potential to reduce ASFV replication or infection in vitro [230]. Finally, in an in vitro model of bovine leukaemia virus infection, the TLR7 agonist GS-9620 increased TNF-α and IFN-γ production and decreased syncytium formation in cultured bovine PBMCs [231], indicating veterinary potential for a drug already under active study as an antiviral in humans [232].

It is worth considering that, while TLR activation is a key mechanism in the development of trained innate immunity, these types of interaction are very complex; activation of some pathways may lead to reduced activity in others or reduced overall flexibility in the response. It is noteworthy that the cross-regulation of TLR function is complex, involving changed activity of positive and negative regulators of signalling and transcriptional changes in the focal TLR as well as others that may be expressed in the same cell (as shown in chickens [233] and in other species (e.g [234–236]), ).

### Beneficial phytochemicals for pathogen defence

Plants have formed the backbone of disease treatment strategies for millennia, and nearly 25% of modern pharmaceuticals are estimated to be derived from plant-origin bioactive chemicals (reviewed in [237, 238]). As plant-derived therapeutics continue to be incorporated into human medicine, our growing understanding of their ability to enhance host immune responses has spurred research into phytochemicals as immunomodulatory alternatives to antimicrobials (reviewed in [239, 240]). However, inherent difficulties in plant research slow the pace of phytochemical drug discovery, and approximately 94% of known plants remain phytochemically uncharacterised [237, 241]. The application of plant-derived bioactive compounds to livestock immunomodulation is therefore a spacious field of research in which promising discoveries have recently been reported, but much remains to be investigated.

Phytochemicals are often administered as dietary supplements to enhance feed utilization, promote reproductive health, improve immune response, etc [242]. In fish, for instance, a comprehensive study of several Indian medicinal plants identified phytol as the primary bioactive component of shrub *Justicia adhatoda*, reporting that dietary supplementation in goldfish (*Carassius auratus*) significantly reduced the mortality associated with experimental *Bacillus licheniformis* infection [243]. Reyes-Becerril et al. published several recent studies of phytochemical immunomodulation in longfin yellowtail (*Seriola rivoliana*). In the first, they tested the composition and properties of phenolic compounds in tejocote (*Crataegus mexicana*) fruit pulp, reporting general antioxidant and immunostimulatory activities in cultured leukocytes and in vivo [244]. A similar study showed increased myeloperoxidase and NO production in the skin mucus of fish fed with Cimarrón plum (*Cyrtocarpa edulis*) fruit, with upregulation of proinflammatory cytokines and piscidin genes observed four weeks later [245]; many of these properties were later linked specifically to the peel of *C. edulis* fruit [246].

Turning to livestock, the flavanol quercetin and the polyphenol curcumin were both reported to promote cell migration, reactive oxygen species production, and direct antibacterial activity in bovine milk-isolated neutrophils exposed to *Streptococcus agalactiae* [247]. Kent-Dennis & Klotz also recently tested the immunomodulatory effects of cannabidiol on LPS-stimulated bovine primary ruminal epithelial cells, observing reduced expression of proinflammatory cytokines and genes encoding caspase 4 and CXCL10 [126]. Meanwhile, an in vitro study of PRRSV-infected porcine monocyte-derived macrophages indicated positive immunomodulatory effects by the plant glycoside rutin, which increased expression of type I interferon-regulated genes while downregulating *TNF-α* [248].

In chickens, Sachan et al. conducted an in-depth study of guduchi vine (*Tinospora cordifolia*) stem aqueous extract as an immunomodulator, comparing its activity to CpG ODN in the management of infectious bursal disease (IBD) in young chicks [249]. Both agents, either alone or in combination, increased IFN-γ and interleukin levels in the PBMCs of specific pathogen-free chicks infected with virulent IBD virus and significantly reduced mortality rates compared to controls [249]. Dietary supplementation with the apple-derived flavonoid phloretin exhibited a protective effect against the necrotic enteritis associated with co-occurring coccidiosis and *Clostridium perfringens* infection in broiler chickens – positive impacts were noted on growth performance, intestinal integrity, oxidative stress, and cytokine production in vivo [250]. Finally, Paradowska et al. reviewed a number of chicken cell culture models (including PBMCs, intestinal epithelial cells, and various immortalised cell lines) and organ cultures for studying the effects of bioactive phytochemicals in vitro and ex vivo [251].

### Nanoparticles

The term “nanoparticles” encompasses an extremely diverse range of particles with a diameter of ~ 1–100 nm and composed of organic, inorganic, polymeric, hybrid, or composite materials [252]. Their many applications include (but are not limited to) drug delivery (e.g., via encapsulation within nanoparticles), targeted immune activation, and vaccination [253–255]. The adaptability, tunability, and basic properties (e.g., high surface area and controllable size) of nanoparticles have attracted much research attention in recent years, particularly in the context of precision medicine in humans ([256]; reviewed in [253, 257]). These technologies have begun to be applied to veterinary medicine as well [258, 259]. Many of these applications centre around vaccine delivery and adjuvant formulation, which are beyond the scope of this paper but have been extensively reviewed elsewhere (e.g [252, 258, 260]), .

Nanoparticles can be used to deliver antimicrobials that could otherwise suffer from toxicity and/or targeting difficulties [261] - e.g., encapsulating silver ions within polyethylene glycol polymer nanoparticles to facilitate ion delivery across bacterial biofilms [262]. Fei et al. used *Bombyx mori* silk fibroin/silver composite nanoparticles to kill *Staphylococcus aureus* and disrupt biofilm formation [263], and veterinary applications have been proposed based on silk nanoparticles’ biocompatibility and many modifiable sites [264]. Nanoparticles can also deliver traditional antibiotics at lower doses to reduce the risks of overtreatment. Yadav et al., for example, used ciprofloxacin-loaded chitosan nanoparticles in vitro against *E. coli* and *S. aureus* isolated from clinical bovine mastitis milk samples, reporting a 50% lower minimum inhibitory concentration compared to the drug alone [265]. However, the delivery system remains to be tested in vivo. Additionally, nanoparticles have been used to aid the delivery and release of dietary supplements and probiotics, where the properties of the encapsulating particle can improve membrane penetration and gastrointestinal absorption (reviewed in [258]).

Interest in metal nanoparticles specifically as veterinary therapeutics initially centred on their direct antimicrobial activity [266], but more recent findings have illustrated additional indirect immunomodulatory effects that improve animal responses to pathogenic infection. Dietary supplementation of broiler chickens with 20 ppm of zinc oxide nanoparticles reduced mortality due to experimentally induced *Eimeria* coccidiosis from approximately 12% to 6%, comparable to treatment with the antiprotozoal diclazuril [267]. Concurrent improvements in growth performance, lesion scores, and oxidative stress markers were also observed [267]. Meanwhile, Kumar et al. reported general improvements in immune and oxidative stress markers in zinc oxide nanoparticle-supplemented iridescent shark catfish (*Pangasianodon hypophthalmus*), linking a dosage of 4.0 mg/kg diet to a host of changes in metabolic, antioxidant, and cytokine gene expression patterns that mitigated arsenic, ammonia, and high temperature stress [268].

Other metal nanoparticles (e.g., gold and silver formulations) have been tested as antimicrobials in vitro and in rodent models, though environmental and cellular toxicity are persistent concerns and few studies have been translated to veterinary applications. Magnetic iron nanoparticles, for instance, were recently tested in vitro in combination with phytochemical extracts of lemon verbena (*Aloysia citriodora/triphylla*), the Rosaceae *Sarcopoterium spinosum*, and the nettle *Urtica pilulifera* – synergistic effects were reported against wild type and multidrug-resistant *E. coli* and *S. aureus* strains, but further studies will be required to assess safety and efficacy in vivo [269].

The challenges and limitations faced by veterinary nanoparticle technologies vary widely depending on the type of particle (and, if applicable, its payload) under study, but they generally include toxic effects in vivo, insufficiently controllable dosage (e.g., via leaking), and accumulation-induced cytotoxicity [270]. For livestock applications, these risks must be considered not only for the animal but also for the human who may consume its products [270]. The regulation and control of nanomedicine-based therapeutics are also underdeveloped in both humans and animals [261]. As the field of nanoparticle research is brimming with potential materials, bioactive cargos, and combinations thereof, the most pressing issues facing the field from a veterinary perspective are the comprehensive testing of promising in vitro candidates in vivo and the increasing development of standardised workflows that allow reproducible comparison and validation in the field.

## Conclusions

The development of livestock immunomodulators as alternatives to antimicrobials builds on simultaneous progress on multiple fronts, among them next-generation sequencing and transcriptomics; genetic manipulation; physiologically relevant in vitro and ex vivo models; and, on the veterinary side, the immunological impact of the many environmental, behavioural, and pathological stressors to which animals are exposed. Immunomodulator application will also need to contend with the additional complexity introduced by the evolution of immunological differences among livestock species, and studying species-specific differences in immunomodulator responses may in turn benefit the field of comparative immunology. As these lines of research continue, synchronisation and collaboration across these fields will remain especially important for practical application of new potential immunomodulators. The STAR IDAZ immunomodulators roadmap was designed to highlight opportunities for cross-discipline collaboration while identifying critical research gaps requiring additional research and investment. Following on from the above discussion of the research nodes included in the roadmap, some of the most pressing of these remaining gaps are shown in Table 1. These gaps stem from numerous factors including the immunological differences among livestock species; the complexities introduced by environmental, dietary, and animal management factors that have overlapping effects on animal immunology; the current dearth of standardised methodologies for measuring veterinary responses to immunomodulators; and the difficulty of performing large-scale in vivo studies in livestock. In order to support delivery in these and related areas, it is critical that we strategically develop greater strengths in the fields of comparative immunology, functional immunogenetics, comparative physiology, and veterinary immunology.

**Table 1:** Research gaps in livestock immunomodulation

STAR IDAZ roadmap nodes	Associated research gaps
17	❖ Genetic/epigenetic factors behind immunocompetence and animal growth/productivity trade-offs
14–16	❖ Mechanisms of host-pathogen interactions and impacts of different microbes, environments, stressors, farm strategies, etc. ❖ Standardisation of data collection, analysis, and reporting to allow direct comparison of immunomodulators ❖ Impacts of dietary supplements on mucosal immunity and epithelial barrier homeostasis
10–13	❖ Mechanisms of immune system development in early life ❖ Impacts of different stressors and diets on immune response/inflammation ❖ Benefits and risks of reducing inflammation in diseased animals ❖ Species-specific nutrient requirements in sub-optimal sanitary conditions
7–9	❖ Immunomodulators as adjuvants for veterinary vaccines ❖ Means and mechanisms of training innate immunity
1–6	❖ In vitro and ex vivo models that reproduce in vivo immunophysiology ❖ Influence of pro- and anti-inflammatory molecules on receptor expression ❖ Identification and characterisation of immunomodulatory phytochemicals ❖ Species-, breed-, and pathogen-specific mechanisms of immunomodulation via TLR agonists ❖ Interactions among different immunomodulators and nutrients ❖ Routes, frequency, and dosage of immunomodulator administration for maximum effect ❖ Toxicology and residue analyses of immunomodulators intended for use in food-producing animals

We must also be ready to meet the regulatory and marketing challenges inherent in putting a new therapeutic product on the market – as discussed above, the limited successes of commercial livestock immunomodulators illustrate the substantial barriers remaining between research and practice. Despite these difficulties, the field of livestock immunomodulation is poised for rapid progress – the explosion of immunomodulator research over the past two decades has greatly expanded our knowledge of host-pathogen interactions, animal innate immunity, and the mechanisms of many HDPs and TLR agonists. As the economic and veterinary threats posed by AMR and zoonotic infections continue to grow, it is increasingly important that these research avenues continue to be explored and expanded.

## Data Availability

No datasets were generated or analysed during the current study.

## Abbreviations

AMP: Antimicrobial peptide
AMR: Antimicrobial resistance
ASFV: African swine fever virus
ATA: Alternatives to Antimicrobials
BCG: Bacillus Calmette-Guérin
BRD: Bovine respiratory disease
DNA: Deoxyribonucleic acid
EDC: Endocrine-disrupting chemical
EV: Extracellular vesicle
HDP: Host defence peptide
IBD: Infectious bursal disease
IFN: Interferon
IL: Interleukin
IRC: International Research Consortium
LPS: Lipopolysaccharide
NDV: Newcastle disease virus
NO: Nitric oxide
ODN: Oligodeoxynucleotides
PBMC: Peripheral blood mononuclear cell
PRR: Pattern recognition receptor
PRRSV: Porcine reproductive and respiratory syndrome virus
RNA: Ribonucleic acid
TLR: Toll-like receptor
TNF: Tumour necrosis factor

## References

[1] Fiddaman SR, Dimopoulos EA, Lebrasseur O, du Plessis L, Vrancken B, Charlton S, et al. Ancient chicken remains reveal the origins of virulence in Marek’s disease virus. Science. 2023;382:1276–81. 10.1126/science.adg2238. 38096384 10.1126/science.adg2238

[2] Bendrey R, Elkholly D, Fournié G. Conceptualizing emergent animal farming and infectious diseases: a one health framework. Evol Med Public Health. 2025;13:344–54. 10.1093/emph/eoaf029. 41221160 10.1093/emph/eoaf029 PMC12599305

[3] Hayek MN. The infectious disease trap of animal agriculture. Sci Adv. 2022;8:eadd6681. 10.1126/sciadv.add6681. 36322670 10.1126/sciadv.add6681 PMC9629715

[4] Leaman SM, Salas S, Mandrell RE, Suslow TV, Jay-Russell MT, Davis DA. Environmental risk factors in the human pathogen transmission pathways between animal operations and produce crops. Food Prot Trends. 2022;42:362–76. 10.4315/FPT-21-041.

[5] Shanta RN, Akther M, Prodhan MA, Akter SH, Annandale H, Sarker S, et al. Adaptation and outbreak of highly pathogenic avian influenza in dairy cattle: an emerging threat to humans, pets, and peridomestic animals. Pathogens. 2025;14:846. 10.3390/pathogens14090846. 41011746 10.3390/pathogens14090846 PMC12473008

[6] Mallapaty S. Animal diseases leapt to humans when we started keeping livestock. Nature. 2025. https://www.nature.com/articles/d41586-025-02165-x. Accessed 9 Jan 2026. 10.1038/d41586-025-02165-x 40634576

[7] Jones KE, Patel NG, Levy MA, Storeygard A, Balk D, Gittleman JL, et al. Global trends in emerging infectious diseases. Nature. 2008;451:990–3. 10.1038/nature06536. 18288193 10.1038/nature06536 PMC5960580

[8] Taylor LH, Latham SM, Woolhouse MEJ. Risk factors for human disease emergence. Philos Trans R Soc B Biol Sci. 2001;356:983–9. 10.1098/rstb.2001.0888. 10.1098/rstb.2001.0888 PMC1088493 11516376

[9] Adegbeye MJ, Adetuyi BO, Igirigi AI, Adisa A, Palangi V, Aiyedun S, et al. Comprehensive insights into antibiotic residues in livestock products: distribution, factors, challenges, opportunities, and implications for food safety and public health. Food Control. 2024;163:110545. 10.1016/j.foodcont.2024.110545.

[10] Murray GGR, Balmer AJ, Herbert J, Hadjirin NF, Kemp CL, Matuszewska M, et al. Mutation rate dynamics reflect ecological change in an emerging zoonotic pathogen. PLOS Genet. 2021;17:e1009864. 10.1371/journal.pgen.1009864. 34748531 10.1371/journal.pgen.1009864 PMC8601623

[11] Ikhimiukor OO, Odih EE, Donado-Godoy P, Okeke IN. A bottom-up view of antimicrobial resistance transmission in developing countries. Nat Microbiol. 2022;7:757–65. 10.1038/s41564-022-01124-w. 35637328 10.1038/s41564-022-01124-w

[12] Mackie RI, Koike S, Krapac I, Chee-Sanford J, Maxwell S, Aminov RI. Tetracycline residues and tetracycline resistance genes in groundwater impacted by swine production facilities. Anim Biotechnol. 2006;17:157–76. 10.1080/10495390600956953. 17127527 10.1080/10495390600956953

[13] Rose Vineer H, Morgan ER, Hertzberg H, Bartley DJ, Bosco A, Charlier J, et al. Increasing importance of anthelmintic resistance in European livestock: Creation and meta-analysis of an open database. Parasite. 2020;27:69. 10.1051/parasite/2020062. 33277891 10.1051/parasite/2020062 PMC7718593

[14] Vercruysse J, Charlier J, Van Dijk J, Morgan ER, Geary T, Von Samson-Himmelstjerna G, et al. Control of helminth ruminant infections by 2030. Parasitology. 2018;145:1655–64. 10.1017/S003118201700227X. 29415781 10.1017/S003118201700227X

[15] Martin MJ, Thottathil SE, Newman TB. Antibiotics overuse in animal agriculture: a call to action for health care providers. Am J Public Health. 2015;105:2409–10. 10.2105/AJPH.2015.302870. 26469675 10.2105/AJPH.2015.302870 PMC4638249

[16] Bava R, Castagna F, Lupia C, Poerio G, Liguori G, Lombardi R, et al. Antimicrobial resistance in livestock: a serious threat to public health. Antibiotics. 2024;13:551. 10.3390/antibiotics13060551. 38927217 10.3390/antibiotics13060551 PMC11200672

[17] Enshaie E, Nigam S, Patel S, Rai V. Livestock antibiotics use and antimicrobial resistance. Antibiot Basel Switz. 2025;14:621. 10.3390/antibiotics14060621. 10.3390/antibiotics14060621 PMC12189104 40558211

[18] Institute of Medicine and National Research Council. Drugs used in food animals: background and perspectives. the use of drugs in food animals: benefits and risks. Washington, D.C.: National Academies; 1999. 10.17226/5137. 25121246

[19] Antimicrobial Resistance Collaborators. Global burden of bacterial antimicrobial resistance in 2019: a systematic analysis. Lancet Lond Engl. 2022;399:629–55. 10.1016/S0140-6736(21)02724-0. 10.1016/S0140-6736(21)02724-0 PMC8841637 35065702

[20] Schmerold I, van Geijlswijk I, Gehring R. European regulations on the use of antibiotics in veterinary medicine. Eur J Pharm Sci. 2023;189:106473. 10.1016/j.ejps.2023.106473. 37220817 10.1016/j.ejps.2023.106473

[21] Dimuccio MM, Conforti V, Celentano FE, Circella E, Salvaggiulo A, Bozzo G, et al. Regulation of antibiotic use in livestock: European and international strategies to prevent and control antimicrobial resistance and ensure animal welfare. Antibiotics. 2026;15:67. 10.3390/antibiotics15010067. 41594105 10.3390/antibiotics15010067 PMC12837735

[22] Centner TJ. Recent government regulations in the United States seek to ensure the effectiveness of antibiotics by limiting their agricultural use. Environ Int. 2016;94:1–7. 10.1016/j.envint.2016.04.018. 27182666 10.1016/j.envint.2016.04.018

[23] Da Silva RA, Arenas NE, Luiza VL, Bermudez JAZ, Clarke SE. Regulations on the use of antibiotics in livestock production in South America: a comparative literature analysis. Antibiotics. 2023;12:1303. 10.3390/antibiotics12081303. 37627723 10.3390/antibiotics12081303 PMC10451520

[24] Yang D, Dyar OJ, Yin J, Ma W, Sun Q, Lundborg CS. Antimicrobial resistance in China across human, animal, and environment sectors – a review of policy documents using a governance framework. Lancet Reg Health – West Pac. 2024;48. 10.1016/j.lanwpc.2024.101111. 10.1016/j.lanwpc.2024.101111 PMC11214315 38948912

[25] Alabi MA, Chenia HY, Lin J. Antibiotic use in livestock: a driver of resistance in Africa and the path to safer alternatives. MicrobiologyOpen. 2025;14:e70122. 10.1002/mbo3.70122. 41214790 10.1002/mbo3.70122 PMC12602276

[26] Mbugua S, Mhone AL, Morang’a A, Gathura P, Onono J, Muloi DM, et al. Veterinary antimicrobial use legislation: a comparative policy analysis of Kenya and Denmark. One Health. 2025;21:101260. 10.1016/j.onehlt.2025.101260. 41283068 10.1016/j.onehlt.2025.101260 PMC12630091

[27] Malik H, Singh R, Kaur S, Dhaka P, Bedi JS, Gill JPS, et al. Review of antibiotic use and resistance in food animal production in WHO South-East Asia Region. J Infect Public Health. 2023;16:172–82. 10.1016/j.jiph.2023.11.002. 37977981 10.1016/j.jiph.2023.11.002

[28] Liu B, Wang W, Deng Z, Ma C, Wang N, Fu C, et al. Antibiotic governance and use on commercial and smallholder farms in eastern China. Front Vet Sci. 2023;10:1128707. 10.3389/fvets.2023.1128707. 37008359 10.3389/fvets.2023.1128707 PMC10065158

[29] Mikecz O, Pica-Ciamarra U, Felis A, Nizeyimana G, Okello P, Brunelli C. Data on antimicrobial use in livestock: Lessons from Uganda. One Health. 2020;10:100165. 10.1016/j.onehlt.2020.100165. 33117878 10.1016/j.onehlt.2020.100165 PMC7582197

[30] Reardon S. Antibiotic use in farming set to soar despite drug-resistance fears. Nature. 2023;614:397. 10.1038/d41586-023-00284-x. 36747098 10.1038/d41586-023-00284-x

[31] Mulchandani R, Wang Y, Gilbert M, Boeckel TPV. Global trends in antimicrobial use in food-producing animals: 2020 to 2030. PLOS Glob Public Health. 2023;3:e0001305. 10.1371/journal.pgph.0001305. 36963007 10.1371/journal.pgph.0001305 PMC10021213

[32] Matheou A, Abousetta A, Pascoe AP, Papakostopoulos D, Charalambous L, Panagi S, et al. Antibiotic use in livestock farming: a driver of multidrug resistance? Microorganisms. 2025;13:779. 10.3390/microorganisms13040779. 40284616 10.3390/microorganisms13040779 PMC12029767

[33] Lin B, Yan S, Zhen B. A machine learning method for predicting molecular antimicrobial activity. Sci Rep. 2025;15:6559. 10.1038/s41598-025-91190-x. 39994442 10.1038/s41598-025-91190-x PMC11850884

[34] Entrican G, Charlier J, Dalton L, Messori S, Sharma S, Taylor R, et al. Construction of generic roadmaps for the strategic coordination of global research into infectious diseases of animals and zoonoses. Transbound Emerg Dis. 2021;68:1513–20. 10.1111/tbed.13821. 32896967 10.1111/tbed.13821

[35] STAR IDAZ IRC. Antimicrobial resistance and alternatives to antimicrobials. 2024. https://www.star-idaz.net/priority-topic/amr-and-innovative-alternatives-to-antibiotics/#roadmap. Accessed 15 Jan 2026.

[36] Douglass T, Anderson A. The emerging immunisation social order: a history of vaccines. Vaccines in Society. Cham: Palgrave Macmillan; 2024. pp. 11–29. 10.1007/978-3-031-61269-5_2.

[37] Bazin H. A brief history of the prevention of infectious diseases by immunisations. Comp Immunol Microbiol Infect Dis. 2003;26:293–308. 10.1016/S0147-9571(03)00016-X. 12818618 10.1016/S0147-9571(03)00016-X

[38] McGill JL, Loving CL, Kehrli ME. Future of immune modulation in animal agriculture. Annu Rev Anim Biosci. 2025;13:255–75. 10.1146/annurev-animal-111523-102209. 39159206 10.1146/annurev-animal-111523-102209

[39] Blecha F. Immunomodulators for Prevention and Treatment of Infectious Diseases in Food-Producing Animals. Vet Clin North Am Food Anim Pract. 2001;17:621–33. 10.1016/S0749-0720(15)30010-4. 11692512 10.1016/s0749-0720(15)30010-4

[40] Lombard M, Pastoret P-P, Moulin A-M. A brief history of vaccines and vaccination. Rev Sci Tech Int Off Epizoot. 2007;26:29–48. 10.20506/rst.26.1.1724. 10.20506/rst.26.1.1724 17633292

[41] McCulloch TR, Wells TJ, Souza-Fonseca-Guimaraes F. Towards efficient immunotherapy for bacterial infection. Trends Microbiol. 2022;30:158–69. 10.1016/j.tim.2021.05.005. 34253452 10.1016/j.tim.2021.05.005

[42] Fenger CK. Limitations to veterinary applications of new technologies in treatment and diagnostics. Vet Clin North Am Equine Pract. 2001;17:389–94. 10.1016/s0749-0739(17)30069-x. 15658183 10.1016/s0749-0739(17)30069-x

[43] Mag P, Nemes-Terényi M, Jerzsele Á, Mátyus P. Some aspects and convergence of human and veterinary drug repositioning. Molecules. 2024;29:4475. 10.3390/molecules29184475. 39339469 10.3390/molecules29184475 PMC11433938

[44] Ahmed I, Kasraian K. Pharmaceutical challenges in veterinary product development. Adv Drug Deliv Rev. 2002;54:871–82. 10.1016/s0169-409x(02)00074-1. 12363436 10.1016/s0169-409x(02)00074-1

[45] Dewsbury DMA, Renter DG, Bradford BJ, DeDonder KD, Mellencamp M, Cernicchiaro N. The application, value, and impact of outcomes research in animal health and veterinary medicine. Front Vet Sci. 2022;9:972057. 10.3389/fvets.2022.972057. 36524226 10.3389/fvets.2022.972057 PMC9744767

[46] Sartini I, Giorgi M. Veterinary pharmacology: a world almost unexplored with huge potential. Vet Anim Sci. 2022;16:100251. 10.1016/j.vas.2022.100251. 35462876 10.1016/j.vas.2022.100251 PMC9020083

[47] Wang Y, Wang J, Zhang L, Han S, Pan X, Wen H, et al. Veterinary drug residues in food chains: sources, exposure pathways, health impacts, mitigation, and safety assurance. Foods. 2026;15:840. 10.3390/foods15050840. 41829113 10.3390/foods15050840 PMC12984630

[48] Nguyen DH, Nguyen TS, Le THH, Nguyen QU, Le Bui N, Chu DT, et al. Evaluation of the safety and immune stimulatory effects of multi-strain Lab Mix product on laboratory animals. Heliyon. 2024;10:e24691. 10.1016/j.heliyon.2024.e24691. 38304811 10.1016/j.heliyon.2024.e24691 PMC10831734

[49] Van de Walle GR, Harman RM. Contributions of large and agricultural animal models to immunology. J Immunol. 2025;214:2494–503. 10.1093/jimmun/vkaf119. 41169229 10.1093/jimmun/vkaf119 PMC12576136

[50] USDA. Summary of Studies Supporting USDA Product Licensure. USDA APHIS. 2025. https://www.aphis.usda.gov/aphis/ourfocus/animalhealth/veterinary-biologics/product-summaries/vet-label-data/daadba18-6af1-4da6-91d5-e8aa3ee1c836

[51] Ons E, Van Brussel L, Lane S, King V, Et A. Efficacy of a Parapoxvirus ovis-based immunomodulator against equine herpesvirus type 1 and Streptococcus equi equi infections in horses. Vet Microbiol. 2014. 10.1016/j.vetmic.2014.07.015. 25153651 10.1016/j.vetmic.2014.07.015

[52] Khan MZ, Ji Y, Fan X, Liu Y, Liu W, Wang C. Equine herpesvirus infections: treatment progress and challenges in horses and donkeys. Vet Sci. 2025;12:1082. 10.3390/vetsci12111082. 41295720 10.3390/vetsci12111082 PMC12656896

[53] Raue R. Zylexis^®^ A new approach for the prophylaxis and treatment of different diseases in horses. in: proceedings of the European Veterinary Conference - Voorjaarsdagen. Amsterdam. 2007. https://www.ivis.org/library/evc/evc-voorjaarsdagen-amsterdam-2007

[54] Nickell JS, Keil DJ, Settje TL, Lechtenberg KF, Singu V, Woolums AR. Efficacy and safety of a novel DNA immunostimulant in cattle. Bov Pract. 2016;9–20. 10.21423/bovine-vol50no1p9-20.

[55] USDA. Summary of Studies supporting USDA Product Licensure. USDA APHIS. 2021. https://www.aphis.usda.gov/print/pdf/node/4543

[56] Oliveira MXS, McGee DD, Brett JA, Larson JE, Stone AE. Evaluation of production parameters and health of dairy cows treated with pegbovigrastim in the transition period. Prev Vet Med. 2020;176:104931. 10.1016/j.prevetmed.2020.104931. 32135413 10.1016/j.prevetmed.2020.104931

[57] Emam M, Mehrabani-Yeganeh H, Barjesteh N, Nikbakht G, Thompson-Crispi K, Charkhkar S, et al. The influence of genetic background versus commercial breeding programs on chicken immunocompetence. Poult Sci. 2014;93:77–84. 10.3382/ps.2013-03475. 24570426 10.3382/ps.2013-03475

[58] Marzouk E, Alajaji AI. Preventive immunology for livestock and zoonotic infectious diseases in the one health era: from mechanistic insights to innovative interventions. Vet Sci. 2025;12:1014. 10.3390/vetsci12101014. 41150154 10.3390/vetsci12101014 PMC12568155

[59] Emam M, Livernois A, Paibomesai M, Atalla H, Mallard B. Genetic and epigenetic regulation of immune response and resistance to infectious diseases in domestic ruminants. Vet Clin North Am Food Anim Pract. 2019;35:405–29. 10.1016/j.cvfa.2019.07.002. 31590895 10.1016/j.cvfa.2019.07.002

[60] Boehm T. Understanding vertebrate immunity through comparative immunology. Nat Rev Immunol. 2025;25:141–52. 10.1038/s41577-024-01083-9. 39317775 10.1038/s41577-024-01083-9

[61] Guzman E, Montoya M. Contributions of farm animals to immunology. Front Vet Sci. 2018;5. 10.3389/fvets.2018.00307. 10.3389/fvets.2018.00307 PMC6292178 30574508

[62] Lamas-Toranzo I, Guerrero-Sánchez J, Miralles-Bover H, Alegre-Cid G, Pericuesta E, Bermejo-Álvarez P. CRISPR is knocking on barn door. Reprod Domest Anim Zuchthyg. 2017;52(Suppl 4):39–47. 10.1111/rda.13047. 10.1111/rda.13047 29052327

[63] Haskell MJ, Simm G, Turner SP. Genetic selection for temperament traits in dairy and beef cattle. Front Genet. 2014;5. 10.3389/fgene.2014.00368. 10.3389/fgene.2014.00368 PMC4204639 25374582

[64] Hu G, Do DN, Gray J, Miar Y. Selection for favorable health traits: a potential approach to cope with diseases in farm animals. Anim Open Access J MDPI. 2020;10:1717. 10.3390/ani10091717. 10.3390/ani10091717 PMC7552752 32971980

[65] Laghouaouta H, Fraile LJ, Pena RN. Selection for resilience in livestock production systems. Int J Mol Sci. 2024;25:13109. 10.3390/ijms252313109. 39684818 10.3390/ijms252313109 PMC11641722

[66] Zou A, Nadeau K, Wang PW, Lee JY, Guttman DS, Sharif S, et al. Accumulation of genetic variants associated with immunity in the selective breeding of broilers. BMC Genet. 2020;21:5. 10.1186/s12863-020-0807-z. 31952471 10.1186/s12863-020-0807-z PMC6969402

[67] Berghof TVL. Selective breeding on natural antibodies in chickens. 2018. 10.18174/426278

[68] Berghof TVL, Matthijs MGR, Arts JAJ, Bovenhuis H, Dwars RM, van der Poel JJ, et al. Selective breeding for high natural antibody level increases resistance to avian pathogenic *Escherichia coli* (APEC) in chickens. Dev Comp Immunol. 2019;93:45–57. 10.1016/j.dci.2018.12.007. 30579935 10.1016/j.dci.2018.12.007

[69] Samuel FU. Breeding program protocols for improving parasite resistance and resilience in small ruminants. Huntsville, Alabama: Alabama A&M University. 2025.

[70] Kasimanickam R, Ferreira JCP, Kastelic J, Kasimanickam V. Application of genomic selection in beef cattle disease prevention. Animals. 2025;15:277. 10.3390/ani15020277. 39858277 10.3390/ani15020277 PMC11759163

[71] Schiller K, Monk JE, Lee C, Horback K. Associations between immune competence phenotype and stress response in sheep. Front Anim Sci. 2023;4. 10.3389/fanim.2023.1160202.

[72] van der Most PJ, de Jong B, Parmentier HK, Verhulst S. Trade-off between growth and immune function: a meta-analysis of selection experiments. Funct Ecol. 2011;25:74–80. 10.1111/j.1365-2435.2010.01800.x.

[73] Vlasova AN, Saif LJ. Bovine immunology: implications for dairy cattle. Front Immunol. 2021;12:643206. 10.3389/fimmu.2021.643206. 34267745 10.3389/fimmu.2021.643206 PMC8276037

[74] Bronzo V, Lopreiato V, Riva F, Amadori M, Curone G, Addis MF, et al. The role of innate immune response and microbiome in resilience of dairy cattle to disease: the Mastitis model. Anim Open Access J MDPI. 2020;10:1397. 10.3390/ani10081397. 10.3390/ani10081397 PMC7459693 32796642

[75] Zerjal T, Härtle S, Gourichon D, Guillory V, Bruneau N, Laloë D, et al. Assessment of trade-offs between feed efficiency, growth-related traits, and immune activity in experimental lines of layer chickens. Genet Sel Evol. 2021;53:44. 10.1186/s12711-021-00636-z. 33957861 10.1186/s12711-021-00636-z PMC8101249

[76] Dadfar M-J, Torshizi RV, Maghsoudi A, Ehsani A, Masoudi AA. Trade-off between feed efficiency and immunity in specialized high-performing chickens. Poult Sci. 2023;102:102703. 10.1016/j.psj.2023.102703. 37141810 10.1016/j.psj.2023.102703 PMC10311186

[77] Vinkler M, Fiddaman SR, Těšický M, O’Connor EA, Savage AE, Lenz TL, et al. Understanding the evolution of immune genes in jawed vertebrates. J Evol Biol. 2023;36:847–73. 10.1111/jeb.14181. 37255207 10.1111/jeb.14181 PMC10247546

[78] Levy H, Fiddaman SR, Vianna JA, Noll D, Clucas GV, Sidhu JKH, et al. Evidence of pathogen-induced immunogenetic selection across the large geographic range of a wild seabird. Mol Biol Evol. 2020;37:1708–26. 10.1093/molbev/msaa040. 32096861 10.1093/molbev/msaa040 PMC7253215

[79] Fiddaman SR, Vinkler M, Spiro SG, Levy H, Emerling CA, Boyd AC, et al. Adaptation and cryptic pseudogenization in penguin toll-like receptors. Mol Biol Evol. 2022;39:msab354. 10.1093/molbev/msab354. 34897511 10.1093/molbev/msab354 PMC8788240

[80] Smith J, Alfieri JM, Anthony N, Arensburger P, Athrey GN, Balacco J, et al. Fourth report on chicken genes and chromosomes 2022. Cytogenet Genome Res. 2023;162:405–528. 10.1159/000529376. 10.1159/000529376 PMC11835228 36716736

[81] O’Donoghue S, Waters SM, Morris DW, Earley B. A Comprehensive review: bovine respiratory disease, current insights into epidemiology, diagnostic challenges, and vaccination. Vet Sci. 2025;12:778. 10.3390/vetsci12080778. 40872728 10.3390/vetsci12080778 PMC12390213

[82] Russell JN, Kos D, Yacoub E, Sies AN, Warr B, Jelinski M, et al. Enhanced metagenomic surveillance for bovine respiratory disease pathogens and antimicrobial resistance by hybridization capture sequencing. Appl Environ Microbiol. 2025;91:e00977–25. 10.1128/aem.00977-25. 40928217 10.1128/aem.00977-25 PMC12542742

[83] Freeman CN, Herman EK, Abi Younes J, Ramsay DE, Erikson N, Stothard P, et al. Evaluating the potential of third generation metagenomic sequencing for the detection of BRD pathogens and genetic determinants of antimicrobial resistance in chronically ill feedlot cattle. BMC Vet Res. 2022;18:211. 10.1186/s12917-022-03269-6. 35655189 10.1186/s12917-022-03269-6 PMC9161498

[84] Johnston D, Earley B, McCabe M, Blackshields G, Lemon K, Duffy C, et al. Application of next generation sequencing for the elucidation of genes and pathways involved in the host response to bovine respiratory syncytial virus. Access Microbiol. 2019;1:605. 10.1099/acmi.ac2019.po0367.

[85] Johnston D, Earley B, Cormican P, Murray G, Kenny DA, Waters SM, et al. Illumina MiSeq 16S amplicon sequence analysis of bovine respiratory disease associated bacteria in lung and mediastinal lymph node tissue. BMC Vet Res. 2017;13:118. 10.1186/s12917-017-1035-2. 28464950 10.1186/s12917-017-1035-2 PMC5414144

[86] O’Donoghue S, Waters SM, Morris DW, Earley B. A comprehensive review: molecular diagnostics and multi-omics approaches to understanding bovine respiratory disease. Vet Sci. 2025;12. 10.3390/vetsci12111095. 10.3390/vetsci12111095 PMC12656982 41295733

[87] Fitzgerald KA, Kagan JC. Toll-like receptors and the control of immunity. Cell. 2020;180:1044–66. 10.1016/j.cell.2020.02.041. 32164908 10.1016/j.cell.2020.02.041 PMC9358771

[88] Werling D, Coffey TJ. Pattern recognition receptors in companion and farm animals – the key to unlocking the door to animal disease? Vet J Lond Engl 1997. 2007;174:240–51. 10.1016/j.tvjl.2006.10.010. 10.1016/j.tvjl.2006.10.010 PMC7110490 17137812

[89] Oboge H, Riitho V, Nyamai M, Omondi GP, Lacasta A, Githaka N, et al. Safety and efficacy of toll-like receptor agonists as therapeutic agents and vaccine adjuvants for infectious diseases in animals: a systematic review. Front Vet Sci. 2024;11. 10.3389/fvets.2024.1428713. 10.3389/fvets.2024.1428713 PMC11442433 39355141

[90] Smith AL, Fiddaman SR. Pattern recognition receptors. In: Kaspers B, Schat KA, Göbel TW, Vervelde L, editors. Avian immunology. Third Edition. Academic Press. 2022:231–48. 10.1016/B978-0-12-818708-1.00026-9.

[91] Maurić Maljković M, Vlahek I, Piplica A, Ekert Kabalin A, Sušić V, Stevanović V. Prospects of toll-like receptors in dairy cattle breeding. Anim Genet. 2023;54:425–34. 10.1111/age.13325. 37051618 10.1111/age.13325

[92] Rehman MS, Rehman S, Yousaf W, Hassan F, Ahmad W, Liu Q, et al. The potential of toll-like receptors to modulate avian immune system: exploring the effects of genetic variants and phytonutrients. Front Genet. 2021;12. 10.3389/fgene.2021.671235. 10.3389/fgene.2021.671235 PMC8427530 34512716

[93] Smiałek M, Tykałowski B, Stenzel T, Koncicki A. Local immunity of the respiratory mucosal system in chickens and turkeys. Pol J Vet Sci. 2011;14:291–7. 10.2478/v10181-011-0047-2. 21721419 10.2478/v10181-011-0047-2

[94] Chase C, Kaushik RS. Mucosal immune system of cattle: all immune responses begin here. Vet Clin North Am Food Anim Pract. 2019;35:431–51. 10.1016/j.cvfa.2019.08.006. 31590896 10.1016/j.cvfa.2019.08.006 PMC7126126

[95] Lesta A, Marín-García PJ, Llobat L. A comprehensive review of the bovine immune response to pathogens. Int J Mol Sci. 2025;26. 10.3390/ijms26178461. 10.3390/ijms26178461 PMC12429741 40943382

[96] Hannant D. Mucosal immunology: overview and potential in the veterinary species. Vet Immunol Immunopathol. 2002;87:265–7. 10.1016/S0165-2427(02)00051-X. 12072245 10.1016/s0165-2427(02)00051-x

[97] Nochi T, Jansen CA, Toyomizu M, van Eden W. The well-developed mucosal immune systems of birds and mammals allow for similar approaches of mucosal vaccination in both types of animals. Front Nutr. 2018;5. 10.3389/fnut.2018.00060. 10.3389/fnut.2018.00060 PMC6052093 30050906

[98] Smith AL, Powers C, Beal R. The avian enteric immune system in health and disease. In: Kaspers B, Schat KA, Göbel TW, Vervelde L, editors. Avian immunology. Third Edition. Academic Press; 2022. pp. 303–26. 10.1016/B978-0-12-818708-1.00030-0

[99] Hammon HM, Frieten D, Gerbert C, Koch C, Dusel G, Weikard R, et al. Different milk diets have substantial effects on the jejunal mucosal immune system of pre-weaning calves, as demonstrated by whole transcriptome sequencing. Sci Rep. 2018;8:1693. 10.1038/s41598-018-19954-2. 29374218 10.1038/s41598-018-19954-2 PMC5785999

[100] Sordillo LM. Nutritional strategies to optimize dairy cattle immunity. J Dairy Sci. 2016;99:4967–82. 10.3168/jds.2015-10354. 26830740 10.3168/jds.2015-10354

[101] Wang M, Ibeagha-Awemu EM. Impacts of epigenetic processes on the health and productivity of livestock. Front Genet. 2021;11:613636. 10.3389/fgene.2020.613636. 33708235 10.3389/fgene.2020.613636 PMC7942785

[102] Grigalevičiūtė R, Matusevičius P, Plančiūnienė R, Stankevičius R, Radzevičiūtė-Valčiukė E, Balevičiūtė A, et al. Understanding the immunomodulatory effects of bovine colostrum: insights into IL-6/IL-10 axis-mediated inflammatory control. Vet Sci. 2023;10. 10.3390/vetsci10080519. 10.3390/vetsci10080519 PMC10458264 37624306

[103] Hadiuzzaman M, Moniruzzaman M, Shahjahan M, Bai SC, Min T, Hossain Z. β-glucan: mode of action and its uses in fish immunomodulation. Front Mar Sci. 2022;9. 10.3389/fmars.2022.905986.

[104] Myhill LJ, Stolzenbach S, Hansen TVA, Skovgaard K, Stensvold CR, Andersen LO, et al. Mucosal barrier and Th2 immune responses are enhanced by dietary inulin in pigs infected with trichuris suis. Front Immunol. 2018;9. 10.3389/fimmu.2018.02557. 10.3389/fimmu.2018.02557 PMC6237860 30473696

[105] Song C, Wei Q, Shen H, Zhang X, Pan D, Zhang Z, et al. Therapeutic potential of Glycyrrhiza polysaccharides in pseudorabies virus infection: immune modulation, antioxidant activity, and gut microbiota restoration. Front Vet Sci. 2025;12. 10.3389/fvets.2025.1679013. 10.3389/fvets.2025.1679013 PMC12575151 41180235

[106] Atuahene D, Sam BA, Idan F, Sana SS, Knop R, Suthar T, et al. Probiotics, prebiotics, and synbiotics in pigs and poultry: a review of gut health, performance, and environmental outcomes. Vet Sci. 2025;12. 10.3390/vetsci12111054. 10.3390/vetsci12111054 PMC12656940 41295692

[107] Chowdhury MR, Hassan M, Shimosato T. Gut health management in livestock: roles of probiotics, prebiotics, and synbiotics in growth, immunity, and microbiota modulation. Vet Res Commun. 2025;49:361. 10.1007/s11259-025-10927-1. 41114910 10.1007/s11259-025-10927-1 PMC12537595

[108] Gomez DE, Galvão KN, Rodriguez-Lecompte JC, Costa MC. The cattle microbiota and the immune system: an evolving field. Vet Clin North Am Food Anim Pract. 2019;35:485–505. 10.1016/j.cvfa.2019.08.002. 31590899 10.1016/j.cvfa.2019.08.002

[109] Kober AKMH, Riaz Rajoka MS, Mehwish HM, Villena J, Kitazawa H. Immunomodulation potential of probiotics: a novel strategy for improving livestock health, immunity, and productivity. Microorganisms. 2022;10:388. 10.3390/microorganisms10020388. 35208843 10.3390/microorganisms10020388 PMC8878146

[110] Yue T, Lu Y, Ding W, Xu B, Zhang C, Li L, et al. The role of probiotics, prebiotics, synbiotics, and postbiotics in livestock and poultry gut health: a review. Metabolites. 2025;15:478. 10.3390/metabo15070478. 40710579 10.3390/metabo15070478 PMC12300891

[111] Ng’onga L, Amoah K, Chen H, Huang Y, Wang B, Shija VM, et al. The metabolism and antioxidant properties of probiotics and prebiotics in fish: a review. Front Mar Sci. 2025;12. 10.3389/fmars.2025.1622474.

[112] Caipang CMA, Lazado CC. Nutritional impacts on fish mucosa: immunostimulants, pre- and probiotics. In: Beck BH, Peatman E, editors. Mucosal Health in Aquaculture. Academic. 2015:211–72. 10.1016/B978-0-12-417186-2.00009-1.

[113] Yu Z, Cantet JM, Nair MRR, Dia VP, Ferguson AB, Cifuentes F, et al. A proinflammatory immune response underlies impairment of intestinal barrier function in Holstein calves exposed to heat stress. J Dairy Sci. 2025;108:11697–706. 10.3168/jds.2025-26930. 40818668 10.3168/jds.2025-26930

[114] Kvamme BO, Gadan K, Finne-Fridell F, Niklasson L, Sundh H, Sundell K, et al. Modulation of innate immune responses in Atlantic salmon by chronic hypoxia-induced stress. Fish Shellfish Immunol. 2013;34:55–65. 10.1016/j.fsi.2012.10.006. 23085636 10.1016/j.fsi.2012.10.006

[115] Parra D, Reyes-Lopez FE, Tort L. Mucosal immunity and B Cells in teleosts: effect of vaccination and stress. Front Immunol. 2015;6:354. 10.3389/fimmu.2015.00354. 26236311 10.3389/fimmu.2015.00354 PMC4502357

[116] Sundh H, Kvamme BO, Fridell F, Olsen RE, Ellis T, Taranger GL, et al. Intestinal barrier function of Atlantic salmon (Salmo salar L.) post smolts is reduced by common sea cage environments and suggested as a possible physiological welfare indicator. BMC Physiol. 2010;10:22. 10.1186/1472-6793-10-22. 21062437 10.1186/1472-6793-10-22 PMC2992494

[117] Abdul Kari Z. Nutritional immunomodulation in aquaculture: functional nutrients, stress resilience, and sustainable health strategies. Aquac Int. 2025;33:441. 10.1007/s10499-025-02122-5.

[118] Okumura R, Takeda K. The role of the mucosal barrier system in maintaining gut symbiosis to prevent intestinal inflammation. Semin Immunopathol. 2025;47:2. 10.1007/s00281-024-01026-5. 10.1007/s00281-024-01026-5 PMC11599372 39589551

[119] Ayalew H, Xu C, Adane A, Sanchez ALB, Li S, Wang J, et al. Ontogeny and function of the intestinal epithelial and innate immune cells during early development of chicks: to explore in ovo immunomodulatory nutrition. Poult Sci. 2025;104:104607. 10.1016/j.psj.2024.104607. 39693955 10.1016/j.psj.2024.104607 PMC11720616

[120] Villena J, Aso H, Rutten VPMG, Takahashi H, van Eden W, Kitazawa H. Immunobiotics for the bovine host: their interaction with intestinal epithelial cells and their effect on antiviral immunity. Front Immunol. 2018;9:326. 10.3389/fimmu.2018.00326. 29599767 10.3389/fimmu.2018.00326 PMC5863502

[121] Karaffová V, Kiššová Z, Tóthová C, Tráj P, Mackei M, Mátis G. Limosilactobacillus reuteri B1/1 modulated the intestinal immune response in preventing Salmonella Enteritidis PT4 infection in a chicken ileal explant model. Vet Res Commun. 2024;49:32. 10.1007/s11259-024-10609-4. 39579331 10.1007/s11259-024-10609-4 PMC11585517

[122] Piper HK, Wiese MJ, Poss MJ. Isolation, establishment, and characterization of immortalized ileum bovine intestinal epithelial cell line. Sci Rep. 2025;15:26652. 10.1038/s41598-025-12386-9. 40695943 10.1038/s41598-025-12386-9 PMC12284070

[123] Danev N, Harman RM, Sipka AS, Oliveira L, Huntimer L, Van de Walle GR. The secretomes of bovine mammary epithelial cell subpopulations differentially modulate macrophage function. Vet Q. 2025;45:1–14. 10.1080/01652176.2025.2463338. 39921381 10.1080/01652176.2025.2463338 PMC11809179

[124] Crawford CK, Lopez Cervantes V, Quilici ML, Armién AG, Questa M, Matloob MS, et al. Inflammatory cytokines directly disrupt the bovine intestinal epithelial barrier. Sci Rep. 2022;12:14578. 10.1038/s41598-022-18771-y. 36028741 10.1038/s41598-022-18771-y PMC9418144

[125] Kowsaw R, Hambruch N, Liu J, Shimizu T, Pfarrer C, Miyamoto A. Regulation of innate immune function in bovine oviduct epithelial cells in culture: the homeostatic role of epithelial cells in balancing Th1/Th2 response. J Reprod Dev. 2013;59:470–8. 10.1262/jrd.2013-036. 23800958 10.1262/jrd.2013-036 PMC3934114

[126] Kent-Dennis C, Klotz JL. Immunomodulation by cannabidiol in bovine primary ruminal epithelial cells. BMC Vet Res. 2023;19:208. 10.1186/s12917-023-03756-4. 37845710 10.1186/s12917-023-03756-4 PMC10577946

[127] Shira EB, Friedman A. Innate immune functions of avian intestinal epithelial cells: Response to bacterial stimuli and localization of responding cells in the developing avian digestive tract. PLoS ONE. 2018;13:e0200393. 10.1371/journal.pone.0200393. 29979771 10.1371/journal.pone.0200393 PMC6034880

[128] Willer T, Kaiser A, Smith A, Rautenschlein S. Morphological and immunological characterization of primary cultured chicken caecal epithelial cells. Eur J Microbiol Immunol. 2024;14:261–71. 10.1556/1886.2024.00053. 10.1556/1886.2024.00053 PMC11393646 38905002

[129] Palladino RA, Colombatto D. Immunometabolism in animal production: building efficiency from health. Anim Front. 2022;12:3–4. 10.1093/af/vfac065.

[130] Monsinjon T, Knigge T. Endocrine disrupters affect the immune system of fish: the example of the European seabass. Fish Shellfish Immunol. 2025;162:110303. 10.1016/j.fsi.2025.110303. 40180203 10.1016/j.fsi.2025.110303

[131] Bobeck EA. Nutrition, and health: companion animal applications. Functional nutrition in livestock and companion animals to modulate the immune response. J Anim Sci. 2020;98:skaa035. 10.1093/jas/skaa035. 32026938 10.1093/jas/skaa035 PMC7053864

[132] Coleman DN, Alharthi AS, Liang Y, Lopes MG, Lopreiato V, Vailati-Riboni M, et al. Multifaceted role of one-carbon metabolism on immunometabolic control and growth during pregnancy, lactation and the neonatal period in dairy cattle. J Anim Sci Biotechnol. 2021;12:27. 10.1186/s40104-021-00547-5. 33536062 10.1186/s40104-021-00547-5 PMC7860211

[133] Eder K, Grundmann SM. Vitamin D in dairy cows: metabolism, status and functions in the immune system. Arch Anim Nutr. 2022;76:1–33. 10.1080/1745039X.2021.2017747. 35249422 10.1080/1745039X.2021.2017747

[134] Ban B, Ki S, Kim S, Park J, Kim S, Jung M, et al. Sucrose supplementation mitigates heat stress-induced alterations in rumen metabolism and immunity in dairy cows. Sci Rep. 2025;15:41758. 10.1038/s41598-025-21840-7. 41271900 10.1038/s41598-025-21840-7 PMC12647585

[135] Zhao X, Wang Y, Wang L, Sun S, Li C, Zhang X, et al. Differences of serum glucose and lipid metabolism and immune parameters and blood metabolomics regarding the transition cows in the antepartum and postpartum period. Front Vet Sci. 2024;11. 10.3389/fvets.2024.1347585. 10.3389/fvets.2024.1347585 PMC10869552 38371596

[136] Loor JJ, Elolimy AA. Immunometabolism in livestock: triggers and physiological role of transcription regulators, nutrients, and microbiota. Anim Front Rev Mag Anim Agric. 2022;12:13–22. 10.1093/af/vfac061. 10.1093/af/vfac061 PMC9564998 36268165

[137] Arfuso F, Minuti A, Liotta L, Giannetto C, Trevisi E, Piccione G, et al. Stress and inflammatory response of cows and their calves during peripartum and early neonatal period. Theriogenology. 2023;196:157–66. 10.1016/j.theriogenology.2022.11.019. 36423510 10.1016/j.theriogenology.2022.11.019

[138] Abuelo A, Mann S, Contreras GA. Metabolic factors at the crossroads of periparturient immunity and inflammation. Vet Clin North Am Food Anim Pract. 2023;39:203–18. 10.1016/j.cvfa.2023.02.012. 37032303 10.1016/j.cvfa.2023.02.012

[139] Rodriguez-Jimenez S, Horst EA, Mayorga EJ, Abeyta MA, Goetz BM, Baumgard LH. Intermittent and increasing intravenous lipopolysaccharide effect on metabolism, inflammation, and production in lactating dairy cows. J Dairy Sci. 2025;108:4283–98. 10.3168/jds.2024-26010. 39824498 10.3168/jds.2024-26010

[140] Trevisi E, Cattaneo L, Piccioli-Cappelli F, Mezzetti M, Minuti A. International symposium on ruminant physiology: the immunometabolism of transition dairy cows from dry-off to early lactation—Lights and shadows. J Dairy Sci. 2025;108:7662–74. 10.3168/jds.2024-25790. 10.3168/jds.2024-25790 39778800

[141] Yang Z, Luo F, Liu G, Luo Z, Ma S, Gao H, et al. Plasma metabolomic analysis reveals the relationship between immune function and metabolic changes in holstein peripartum dairy cows. Metabolites. 2022;12:953. 10.3390/metabo12100953. 36295855 10.3390/metabo12100953 PMC9611258

[142] Zachut M, Tam J, Contreras GA. Modulating immunometabolism in transition dairy cows: the role of inflammatory lipid mediators. Anim Front. 2022;12:37–45. 10.1093/af/vfac062. 36268169 10.1093/af/vfac062 PMC9564993

[143] Wang Z, Wang Q, Tang C, Yuan J, Luo C, Li D, et al. Medium chain fatty acid supplementation improves animal metabolic and immune status during the transition period: a study on dairy cattle. Front Immunol. 2023;14. 10.3389/fimmu.2023.1018867. 10.3389/fimmu.2023.1018867 PMC9911908 36776875

[144] Brown EJ, Vosloo A. The involvement of the hypothalamopituitary-adrenocortical axis in stress physiology and its significance in the assessment of animal welfare in cattle. Onderstepoort J Vet Res. 2017;84:9. 10.4102/ojvr.v84i1.1398. 10.4102/ojvr.v84i1.1398 PMC6238696 28470085

[145] Gouvêa VN, Cooke RF, Marques RS. Impacts of stress-induced inflammation on feed intake of beef cattle. Front Anim Sci. 2022;3. 10.3389/fanim.2022.962748.

[146] Gupta S, Sharma A, Joy A, Dunshea FR, Chauhan SS. The impact of heat stress on immune status of dairy cattle and strategies to ameliorate the negative effects. Anim Open Access J MDPI. 2022;13:107. 10.3390/ani13010107. 10.3390/ani13010107 PMC9817836 36611716

[147] Niu X, Ding Y, Chen S, Gooneratne R, Ju X. Effect of immune stress on growth performance and immune functions of livestock: mechanisms and prevention. Anim Open Access J MDPI. 2022;12:909. 10.3390/ani12070909. 10.3390/ani12070909 PMC8996973 35405897

[148] Lopez BS, Hurley DJ, Giancola S, Giguère S, Hart KA. The effect of cortisol on equine monocyte–derived dendritic cell phenotype and cytokine production. Vet Med Sci. 2024;10:e1333. 10.1002/vms3.1333.

[149] Shabtay A. Combined approaches to reduce stress and improve livestock well-being: a review. Cell Stress Chaperones. 2025;30:100091. 10.1016/j.cstres.2025.100091. 40645440 10.1016/j.cstres.2025.100091 PMC12304687

[150] Cortese VS. Applied immunology and the effects of stress on the immune system. AABP Proc. 2019;46–49. https://bovine-ojs-tamu.tdl.org/AABP/issue/view/174

[151] Tuchscherer M, Kanitz E, Puppe B, Tuchscherer A. Altered immunomodulation by glucocorticoids in neonatal pigs exposed to a psychosocial stressor. Pediatr Res. 2010;68:473–8. 10.1203/PDR.0b013e3181f70f08. 20717071 10.1203/PDR.0b013e3181f70f08

[152] Orihuela A, Review. Management of livestock behavior to improve welfare and production. Anim Int J Anim Biosci. 2021;15. 10.1016/j.animal.2021.100290. Suppl 1:100290. 10.1016/j.animal.2021.100290 34238724

[153] Louchouarn NX, Treves A. Low-stress livestock handling protects cattle in a five-predator habitat. PeerJ. 2023;11:e14788. 10.7717/peerj.14788. 36793893 10.7717/peerj.14788 PMC9924134

[154] Morgado JN, Lamonaca E, Santeramo FG, Caroprese M, Albenzio M, Ciliberti MG. Effects of management strategies on animal welfare and productivity under heat stress: a synthesis. Front Vet Sci. 2023;10:1145610. 10.3389/fvets.2023.1145610. 37008346 10.3389/fvets.2023.1145610 PMC10050400

[155] Grandin T. Handling methods and facilities to reduce stress on cattle. Vet Clin North Am Food Anim Pract. 1998;14:325–41. 10.1016/s0749-0720(15)30257-7. 9704418 10.1016/s0749-0720(15)30257-7

[156] Opgenorth J, Sordillo LM, Lock AL, Gandy JC, VandeHaar MJ. Colostrum supplementation with n-3 fatty acids alters plasma polyunsaturated fatty acids and inflammatory mediators in newborn calves. J Dairy Sci. 2020;103:11676–88. 10.3168/jds.2019-18045. 33041038 10.3168/jds.2019-18045 PMC7544567

[157] Fabjanowska J, Kowalczuk-Vasilev E, Klebaniuk R, Milewski S, Gümüş H. N-3 Polyunsaturated fatty acids as a nutritional support of the reproductive and immune system of cattle—a review. Animals. 2023;13. 10.3390/ani13223589. 10.3390/ani13223589 PMC10668692 38003206

[158] McBride ML, Sanchez NCB, Carroll JA, Broadway PR, Ortiz XO, Collier JL et al. 1128 OmniGen-AF^®^ reduces basal plasma cortisol as well as cortisol release to adrencocorticotropic hormone or corticotrophin releasing hormone and vasopressin in lactating dairy cows under thermoneutral or acute heat stress conditions. J Anim Sci. 2016;94 suppl_5:541–2. 10.2527/jam2016-1128

[159] Marins TN, Gao J, Yang Q, Binda RM, Pessoa CMB, Orellana Rivas RM, et al. Impact of heat stress and a feed supplement on hormonal and inflammatory responses of dairy cows. J Dairy Sci. 2021;104:8276–89. 10.3168/jds.2021-20162. 33865597 10.3168/jds.2021-20162

[160] Zhu HL, Liu YL, Xie XL, Huang JJ, Hou YQ. Effect of L-arginine on intestinal mucosal immune barrier function in weaned pigs after Escherichia coli LPS challenge. Innate Immun. 2013;19:242–52. 10.1177/1753425912456223. 22904165 10.1177/1753425912456223

[161] Yakah W, Ramiro-Cortijo D, Singh P, Brown J, Stoll B, Kulkarni M, et al. Parenteral fish-oil containing lipid emulsions limit initial lipopolysaccharide-induced host immune responses in preterm pigs. Nutrients. 2021;13:205. 10.3390/nu13010205. 33445698 10.3390/nu13010205 PMC7828127

[162] Zhang X-Y, Xu Z-P, Wang W, Cao J-B, Fu Q, Zhao W-X, et al. Vitamin C alleviates LPS-induced cognitive impairment in mice by suppressing neuroinflammation and oxidative stress. Int Immunopharmacol. 2018;65:438–47. 10.1016/j.intimp.2018.10.020. 30388518 10.1016/j.intimp.2018.10.020

[163] Upadhaya SD, Kim JC, Mullan BP, Pluske JR, Kim IH. Vitamin E and omega-3 fatty acids independently attenuate plasma concentrations of proinflammatory cytokines and prostaglandin E2 in Escherichia coli lipopolysaccharide-challenged growing–finishing pigs1. J Anim Sci. 2015;93:2926–34. 10.2527/jas.2014-8330. 26115279 10.2527/jas.2014-8330

[164] Song C, Jiang J, Han X, Yu G, Pang Y. Effect of immunological stress to neuroendocrine and gene expression in different swine breeds. Mol Biol Rep. 2014;41:3569–76. 10.1007/s11033-014-3219-1. 24515387 10.1007/s11033-014-3219-1

[165] Zhou X, Yang Z, Luo Z, Li H, Chen G. Endocrine disrupting chemicals in wild freshwater fishes: species, tissues, sizes and human health risks. Environ Pollut. 2019;244:462–8. 10.1016/j.envpol.2018.10.026. 30366293 10.1016/j.envpol.2018.10.026

[166] Peskova N, Blahova J, Bisphenols. Endocrine disruptors and their impact on fish: a review. Fishes. 2025;10:365. 10.3390/fishes10080365.

[167] Kernen L, Phan A, Bo J, Herzog EL, Huynh J, Segner H, et al. Estrogens as immunotoxicants: 17α-ethinylestradiol exposure retards thymus development in zebrafish (*Danio rerio*). Aquat Toxicol. 2022;242:106025. 10.1016/j.aquatox.2021.106025. 34837781 10.1016/j.aquatox.2021.106025

[168] Paiola M, Knigge T, Picchietti S, Duflot A, Guerra L, Pinto PIS, et al. Oestrogen receptor distribution related to functional thymus anatomy of the European sea bass, *Dicentrarchus labrax*. Dev Comp Immunol. 2017;77:106–20. 10.1016/j.dci.2017.07.023. 28756001 10.1016/j.dci.2017.07.023

[169] Burgos-Aceves MA, Cohen A, Smith Y, Faggio C. Estrogen regulation of gene expression in the teleost fish immune system. Fish Shellfish Immunol. 2016;58:42–9. 10.1016/j.fsi.2016.09.006. 27633675 10.1016/j.fsi.2016.09.006

[170] Leet JK, Greer JB, Richter CA, Iwanowicz LR, Spinard E, McDonald J, et al. Exposure to 17α-ethinylestradiol results in differential susceptibility of largemouth bass (Micropterus salmoides) to bacterial infection. Environ Sci Technol. 2022;56:14375–86. 10.1021/acs.est.2c02250. 36197672 10.1021/acs.est.2c02250 PMC9583602

[171] da Silva RC, Teixeira MP, de Paiva LS, Miranda-Alves L. Environmental health and toxicology: immunomodulation promoted by endocrine-disrupting chemical tributyltin. Toxics. 2023;11. 10.3390/toxics11080696. 10.3390/toxics11080696 PMC10458372 37624201

[172] STAR IDAZ IRC. Roadmap for vaccine development. https://www.star-idaz.net/roadmap/roadmap-for-vaccine-development/. Accessed 30 Jan 2026.

[173] Entrican G, Francis MJ. Applications of platform technologies in veterinary vaccinology and the benefits for one health. Vaccine. 2022;40:2833–40. 10.1016/j.vaccine.2022.03.059. 35382957 10.1016/j.vaccine.2022.03.059

[174] Entrican G, Bredell H, Charlier J, Cunningham AF, Jarvis MA, Wood PR, et al. Opportunities and challenges for the adoption of novel platform technologies to develop veterinary bacterial vaccines. Vaccine. 2025;54:127117. 10.1016/j.vaccine.2025.127117. 40233592 10.1016/j.vaccine.2025.127117

[175] Alzan HF, Mahmoud MS, Suarez CE. Current vaccines, experimental immunization trials, and new perspectives to control selected vector borne blood parasites of veterinary importance. Front Vet Sci. 2024;11. 10.3389/fvets.2024.1484787. 10.3389/fvets.2024.1484787 PMC11602000 39606652

[176] Hoelzer K, Bielke L, Blake DP, Cox E, Cutting SM, Devriendt B, et al. Vaccines as alternatives to antibiotics for food producing animals. Part 1: challenges and needs. Vet Res. 2018;49:64. 10.1186/s13567-018-0560-8. 30060757 10.1186/s13567-018-0560-8 PMC6066911

[177] Hoelzer K, Bielke L, Blake DP, Cox E, Cutting SM, Devriendt B, et al. Vaccines as alternatives to antibiotics for food producing animals. Part 2: new approaches and potential solutions. Vet Res. 2018;49:70. 10.1186/s13567-018-0561-7. 30060759 10.1186/s13567-018-0561-7 PMC6066917

[178] Islam MS, Rahman MT. A comprehensive review on bacterial vaccines combating antimicrobial resistance in poultry. Vaccines. 2023;11. 10.3390/vaccines11030616. 10.3390/vaccines11030616 PMC10051968 36992200

[179] Micoli F, Bagnoli F, Rappuoli R, Serruto D. The role of vaccines in combatting antimicrobial resistance. Nat Rev Microbiol. 2021;19:287–302. 10.1038/s41579-020-00506-3. 33542518 10.1038/s41579-020-00506-3 PMC7861009

[180] Tadesse BT, Keddy KH, Rickett NY, Zhusupbekova A, Poudyal N, Lawley T, et al. Vaccination to reduce antimicrobial resistance burden—data gaps and future research. Clin Infect Dis. 2023;77 Supplement7:S597–607. 10.1093/cid/ciad562. 38118013 10.1093/cid/ciad562 PMC10732565

[181] Pernthaner A, Simpson H, Umair S. Parasite vaccines. In: Metwally S, El Idrissi A, Viljoen G, editors. Veterinary vaccines. John Wiley & Sons, Ltd. 2021:101–11. 10.1002/9781119506287.ch8.

[182] Vekemans J, Hasso-Agopsowicz M, Kang G, Hausdorff WP, Fiore A, Tayler E, et al. Leveraging vaccines to reduce antibiotic use and prevent antimicrobial resistance: a World Health Organization action framework. Clin Infect Dis Off Publ Infect Dis Soc Am. 2021;73:e1011–7. 10.1093/cid/ciab062. 10.1093/cid/ciab062 PMC8366823 33493317

[183] Kurtz J, Andino R, Boraschi D, Contreras-Garduño J, Kachroo A, Khan I, et al. Trained immunity and immune priming in plants and invertebrates. eLife. 2025;14:e106597. 10.7554/eLife.106597. 41347291 10.7554/eLife.106597 PMC12680377

[184] Sharrock J, Sun JC. Innate immunological memory: from plants to animals. Curr Opin Immunol. 2020;62:69–78. 10.1016/j.coi.2019.12.001. 31931432 10.1016/j.coi.2019.12.001 PMC7067670

[185] Báez-Magaña M, Alva-Murillo N, Ochoa-Zarzosa A, López-Meza JE. Trained immunity in farm animals. Vet Res. 2025;56:166. 10.1186/s13567-025-01594-w. 40781647 10.1186/s13567-025-01594-w PMC12335096

[186] Netea MG, Quintin J, van der Meer JWM. Trained immunity: a memory for innate host defense. Cell Host Microbe. 2011;9:355–61. 10.1016/j.chom.2011.04.006. 21575907 10.1016/j.chom.2011.04.006

[187] Yoshimura Y, Nii T, Isobe N. Innate immune training in chickens for improved defense against pathogens: a review. J Poult Sci. 2024;61. 10.2141/jpsa.2024008. 10.2141/jpsa.2024008 PMC10925860 38481975

[188] Verwoolde MB, van den Biggelaar RHGA, de Vries Reilingh G, Arts JAJ, van Baal J, Lammers A, et al. Innate immune training and metabolic reprogramming in primary monocytes of broiler and laying hens. Dev Comp Immunol. 2021;114:103811. 10.1016/j.dci.2020.103811. 32750399 10.1016/j.dci.2020.103811

[189] Schünemann L-M, Schuberth H-J. Non-classical monocytes contribute to innate immune training in cattle. Innate Immun. 2022;28:199–210. 10.1177/17534259221114219. 35876352 10.1177/17534259221114219 PMC9389050

[190] Jia J, Ji W, Xiong N, Lin J, Yang Q. Trained immunity using probiotics and inactivated pathogens enhances resistance to *Salmonella enterica* serovar *Typhimurium* infection by activating the cGAS-STING signal pathway in mice and chickens. J Adv Res. 2026;79:491–504. 10.1016/j.jare.2025.03.011. 40086629 10.1016/j.jare.2025.03.011 PMC12766225

[191] Netea MG, Joosten LAB, Latz E, Mills KHG, Natoli G, Stunnenberg HG, et al. Trained immunity: a program of innate immune memory in health and disease. Science. 2016;352:aaf1098. 10.1126/science.aaf1098. 27102489 10.1126/science.aaf1098 PMC5087274

[192] Blok BA, Arts RJW, van Crevel R, Benn CS, Netea MG. Trained innate immunity as underlying mechanism for the long-term, nonspecific effects of vaccines. J Leukoc Biol. 2015;98:347–56. 10.1189/jlb.5RI0315-096R. 26150551 10.1189/jlb.5RI0315-096R

[193] Guerra-Maupome M, Vang DX, McGill JL. Aerosol vaccination with Bacille Calmette-Guerin induces a trained innate immune phenotype in calves. PLoS ONE. 2019;14:e0212751. 10.1371/journal.pone.0212751. 30794653 10.1371/journal.pone.0212751 PMC6386280

[194] Samuel BER, Diaz FE, Maina TW, Corbett RJ, Tuggle CK, McGill JL. Evidence of innate training in bovine γδ T cells following subcutaneous BCG administration. Front Immunol. 2024;15. 10.3389/fimmu.2024.1423843. 10.3389/fimmu.2024.1423843 PMC11295143 39100669

[195] Saubi C, Meade KG, Travé-Asensio S, Garcia-Fruitós E, Aris A. Immunomodulatory activities of bovine host defense peptides (HDPs): context-dependent roles in inflammation and immune priming. BMC Vet Res. 2026. 10.1186/s12917-025-05258-x. 41535881 10.1186/s12917-025-05258-x PMC12947540

[196] Hong Y, Lee J, Vu TH, Lee S, Lillehoj HS, Hong YH. Chicken avian β-defensin 8 modulates immune response via the mitogen-activated protein kinase signaling pathways in a chicken macrophage cell line. Poult Sci. 2020;99:4174–82. 10.1016/j.psj.2020.05.027. 32867961 10.1016/j.psj.2020.05.027 PMC7598012

[197] Xie K, Su G, Chen D, Yu B, Huang Z, Yu J, et al. The immunomodulatory function of the porcine β-defensin 129: alleviate inflammatory response induced by LPS in IPEC-J2 cells. Int J Biol Macromol. 2021;188:473–81. 10.1016/j.ijbiomac.2021.07.194. 34352320 10.1016/j.ijbiomac.2021.07.194

[198] Finatto AN, Yang C, de Oliveira Costa M. Porcine β-defensin 5 (pBD-5) modulates the inflammatory and metabolic host intestinal response to infection. Sci Rep. 2025;15:7568. 10.1038/s41598-025-90688-8. 40038370 10.1038/s41598-025-90688-8 PMC11880483

[199] Zhou Y, Zhou Q-J, Qiao Y, Chen J, Li M-Y. The host defense peptide β-defensin confers protection against Vibrio anguillarum in ayu, Plecoglossus altivelis. Dev Comp Immunol. 2020;103:103511. 10.1016/j.dci.2019.103511. 31580833 10.1016/j.dci.2019.103511

[200] Salger SA, Cassady KR, Reading BJ, Noga EJ. A diverse family of host-defense peptides (Piscidins) exhibit specialized anti-bacterial and anti-protozoal activities in fishes. PLoS ONE. 2016;11:e0159423. 10.1371/journal.pone.0159423. 27552222 10.1371/journal.pone.0159423 PMC4995043

[201] Wang Y, Wang M, Shan A, Feng X. Avian host defense cathelicidins: structure, expression, biological functions, and potential therapeutic applications. Poult Sci. 2020;99:6434–45. 10.1016/j.psj.2020.09.030. 33248558 10.1016/j.psj.2020.09.030 PMC7704953

[202] van Harten RM, van Woudenbergh E, van Dijk A, Haagsman HP, Cathelicidins. Immunomodulatory antimicrobials vaccines. 2018;6:63. 10.3390/vaccines6030063. 30223448 10.3390/vaccines6030063 PMC6161271

[203] Peng L, Scheenstra MR, van Harten RM, Haagsman HP, Veldhuizen EJA. The immunomodulatory effect of cathelicidin-B1 on chicken macrophages. Vet Res. 2020;51:122. 10.1186/s13567-020-00849-y. 32972448 10.1186/s13567-020-00849-y PMC7517697

[204] Peng L, Lu Y, Tian H, Jia K, Tao Q, Li G, et al. Chicken cathelicidin-2 promotes IL-1β secretion via the NLRP3 inflammasome pathway and serine proteases activity in LPS-primed murine neutrophils. Dev Comp Immunol. 2022;131:104377. 10.1016/j.dci.2022.104377. 35189160 10.1016/j.dci.2022.104377

[205] Kraaij MD, van Dijk A, Scheenstra MR, van Harten RM, Haagsman HP, Veldhuizen EJA. Chicken CATH-2 increases antigen presentation markers on chicken monocytes and macrophages. Protein Pept Lett. 2020;27:60–6. 10.2174/0929866526666190730125525. 31362652 10.2174/0929866526666190730125525 PMC6978643

[206] Goitsuka R, Chen CH, Benyon L, Asano Y, Kitamura D, Cooper MD. Chicken cathelicidin-B1, an antimicrobial guardian at the mucosal M cell gateway. Proc Natl Acad Sci U S A. 2007;104:15063–8. 10.1073/pnas.0707037104. 17827276 10.1073/pnas.0707037104 PMC1986613

[207] Achanta M, Sunkara LT, Dai G, Bommineni YR, Jiang W, Zhang G. Tissue expression and developmental regulation of chicken cathelicidin antimicrobial peptides. J Anim Sci Biotechnol. 2012;3:15. 10.1186/2049-1891-3-15. 22958518 10.1186/2049-1891-3-15 PMC3436658

[208] Liu X, Wang X, Shi X, Wang S, Shao K. The immune enhancing effect of antimicrobial peptide LLv on broilers chickens. Poult Sci. 2024;103:103235. 10.1016/j.psj.2023.103235. 38035471 10.1016/j.psj.2023.103235 PMC10698674

[209] Márton RA, Sebők C, Mackei M, Tráj P, Vörösházi J, Kemény Á, et al. Cecropin A: investigation of a host defense peptide with multifaceted immunomodulatory activity in a chicken hepatic cell culture. Front Vet Sci. 2024;11. 10.3389/fvets.2024.1337677. 10.3389/fvets.2024.1337677 PMC10940386 38496311

[210] Márton RA, Varga O, Vincent NJ, Tráj P, Sebők C, Kemény Á, et al. Effects of cecropin A on cytokine production and tight junction protein expression in chicken ileal explant cultures. Vet Res Commun. 2026;50:104. 10.1007/s11259-025-11046-7. 41537918 10.1007/s11259-025-11046-7 PMC12808300

[211] Márton RA, Tráj P, Mackei M, Sebők C, Vörösházi J, Kemény Á, et al. Exploring the impact of the host defense peptide Pap12-6 on immune response and epithelial integrity in chicken-derived ileal explant cultures. Poult Sci. 2025;104:105376. 10.1016/j.psj.2025.105376. 40466266 10.1016/j.psj.2025.105376 PMC12169739

[212] Ye C, Wan C, Chen J, Li G, Li Y, Wang Y, et al. Cathelicidin CATH-B1 inhibits pseudorabies virus infection via direct interaction and TLR4/JNK/IRF3-mediated interferon activation. J Virol. 2023;97:e0070623. 10.1128/jvi.00706-23. 37314341 10.1128/jvi.00706-23 PMC10373553

[213] Ouyang J, Zhu Y, Hao W, Wang X, Yang H, Deng X, et al. Three naturally occurring host defense peptides protect largemouth bass (*Micropterus salmoides*) against bacterial infections. Aquaculture. 2022;546:737383. 10.1016/j.aquaculture.2021.737383.

[214] Kumar R, Ali SA, Singh SK, Bhushan V, Mathur M, Jamwal S, et al. Antimicrobial peptides in farm animals: an updated review on its diversity, function, modes of action and therapeutic prospects. Vet Sci. 2020;7:206. 10.3390/vetsci7040206. 33352919 10.3390/vetsci7040206 PMC7766339

[215] Bosso A, Di Nardo I, Culurciello R, Palumbo I, Gaglione R, Zannella C, et al. KNR50: a moonlighting bioactive peptide hidden in the C-terminus of bovine casein αS2 with powerful antimicrobial, antibiofilm, antiviral and immunomodulatory activities. Int J Biol Macromol. 2025;311:143718. 10.1016/j.ijbiomac.2025.143718. 40339849 10.1016/j.ijbiomac.2025.143718

[216] Blanco FC, Gravisaco MJ, Bigi MM, García EA, Marquez C, McNeil M, et al. Identifying bacterial and host factors involved in the interaction of mycobacterium bovis with the bovine innate immune cells. Front Immunol. 2021;12. 10.3389/fimmu.2021.674643. 10.3389/fimmu.2021.674643 PMC8319915 34335572

[217] Infantes-Lorenzo JA, Moreno I, Risalde M, de los Á, Roy Á, Villar M, Romero B, et al. Proteomic characterisation of bovine and avian purified protein derivatives and identification of specific antigens for serodiagnosis of bovine tuberculosis. Clin Proteom. 2017;14:36. 10.1186/s12014-017-9171-z. 10.1186/s12014-017-9171-z PMC5669029 29142508

[218] Agulló-Ros I, Burucúa MM, Cheuquepán FA, Domínguez M, Sevilla IA, Martínez R, et al. Heat-inactivated *Mycobacterium bovis* and P22PI protein immunocomplex: Two candidates for use as immunostimulants of innate immune response. Vet Microbiol. 2025;305:110527. 10.1016/j.vetmic.2025.110527. 40279721 10.1016/j.vetmic.2025.110527

[219] Hussen J, Alkuwayti MA, Falemban B, Al-Sukruwah MA, Alhojaily SM, Humam NAA, et al. Immunomodulatory effects of bacterial toll-like receptor ligands on the phenotype and function of milk immune cells in dromedary camel. Biology. 2023;12. 10.3390/biology12020276. 10.3390/biology12020276 PMC9952959 36829554

[220] Kaur A, Baldwin J, Brar D, Salunke DB, Petrovsky N. Toll-like receptor (TLR) agonists as a driving force behind next-generation vaccine adjuvants and cancer therapeutics. Curr Opin Chem Biol. 2022;70:102172. 10.1016/j.cbpa.2022.102172. 35785601 10.1016/j.cbpa.2022.102172

[221] Shao F, Zhu X, Yi M, Gao H, Wu J, Fang R, et al. TLR agonists as adjuvants for viral vaccines: mechanisms, applications, and future directions. Front Microbiol. 2025;16:1740572. 10.3389/fmicb.2025.1740572. 41568029 10.3389/fmicb.2025.1740572 PMC12815852

[222] Bavananthasivam J, Alkie TN, Astill J, Abdul-Careem MF, Wootton SK, Behboudi S, et al. In ovo administration of Toll-like receptor ligands encapsulated in PLGA nanoparticles impede tumor development in chickens infected with Marek’s disease virus. Vaccine. 2018;36:4070–6. 10.1016/j.vaccine.2018.05.091. 29859800 10.1016/j.vaccine.2018.05.091

[223] Chen X, Zhang P, Li P, Wang G, Li J, Wu Y, et al. CpG ODN 1668 as TLR9 agonist mediates humpback grouper (Cromileptes altivelis) antibacterial immune responses. Fish Shellfish Immunol. 2023;138:108839. 10.1016/j.fsi.2023.108839. 37207883 10.1016/j.fsi.2023.108839

[224] Sharma BK, Kakker NK, Bhadouriya S, Chhabra R. Effect of TLR agonist on infections bronchitis virus replication and cytokine expression in embryonated chicken eggs. Mol Immunol. 2020;120:52–60. 10.1016/j.molimm.2020.02.001. 32065987 10.1016/j.molimm.2020.02.001 PMC7112572

[225] Abdul-Cader MS, De Silva Senapathi U, Nagy E, Sharif S, Abdul-Careem MF. Antiviral response elicited against avian influenza virus infection following activation of toll-like receptor (TLR)7 signaling pathway is attributable to interleukin (IL)-1β production. BMC Res Notes. 2018;11:859. 10.1186/s13104-018-3975-4. 30514372 10.1186/s13104-018-3975-4 PMC6280464

[226] Gupta A, Deka P, Kumar S. Resiquimod inhibits Newcastle disease virus replication by modulating host cytokines: an understanding towards its possible therapeutics. Cytokine. 2020;125:154811. 10.1016/j.cyto.2019.154811. 31446178 10.1016/j.cyto.2019.154811

[227] Huang Q, Yang H, Yang D, Hao Y, Yu S, Guo Z, et al. A synthetic toll-like receptor 7 agonist inhibits porcine reproductive and respiratory syndrome virus replication in piglets. Vet Microbiol. 2022;271:109475. 10.1016/j.vetmic.2022.109475. 35660287 10.1016/j.vetmic.2022.109475

[228] Vreman S, Rebel JMJ, McCaffrey J, Ledl K, Arkhipova K, Collins D, et al. Early immune responses in skin and lymph node after skin delivery of Toll-like receptor agonists in neonatal and adult pigs. Vaccine. 2021;39:1857–69. 10.1016/j.vaccine.2021.02.028. 33678451 10.1016/j.vaccine.2021.02.028

[229] de Agricultura M. Pesca y Alimentación. African Swine Fever in wild boars in Spain. 2025. https://rr-europe.woah.org/app/uploads/2025/12/ASF-situation-in-Spain.pdf

[230] Franzoni G, Zinellu S, Razzuoli E, Mura L, De Ciucis CG, De Paolis L, et al. Assessment of the impact of a toll-like receptor 2 agonist synthetic lipopeptide on macrophage susceptibility and responses to African swine fever virus infection. Viruses. 2022;14:2212. 10.3390/v14102212. 36298767 10.3390/v14102212 PMC9610641

[231] Sajiki Y, Konnai S, Okagawa T, Maekawa N, Nakamura H, Kato Y, et al. A TLR7 agonist activates bovine Th1 response and exerts antiviral activity against bovine leukemia virus. Dev Comp Immunol. 2021;114:103847. 10.1016/j.dci.2020.103847. 32888966 10.1016/j.dci.2020.103847

[232] Janssen HLA, Brunetto MR, Kim YJ, Ferrari C, Massetto B, Nguyen A-H, et al. Safety, efficacy and pharmacodynamics of vesatolimod (GS-9620) in virally suppressed patients with chronic hepatitis B. J Hepatol. 2018;68:431–40. 10.1016/j.jhep.2017.10.027. 29104121 10.1016/j.jhep.2017.10.027

[233] Peroval MY, Boyd AC, Young JR, Smith AL. A Critical role for MAPK signalling pathways in the transcriptional regulation of toll like receptors. PLoS ONE. 2013;8:e51243. 10.1371/journal.pone.0051243. 23405061 10.1371/journal.pone.0051243 PMC3566169

[234] Zannetti C, Parroche P, Panaye M, Roblot G, Gruffat H, Manet E, et al. TLR9 transcriptional regulation in response to double-stranded DNA viruses. J Immunol. 2014;193:3398–408. 10.4049/jimmunol.1400249. 25194054 10.4049/jimmunol.1400249

[235] Sominsky L, Sobinoff AP, Jobling MS, Pye V, McLaughlin EA, Hodgson DM. Immune regulation of ovarian development: programming by neonatal immune challenge. Front Neurosci. 2013;7:100. 10.3389/fnins.2013.00100. 23781169 10.3389/fnins.2013.00100 PMC3679471

[236] Ding X, Liang Y, Peng W, Li R, Lin H, Zhang Y, et al. Intracellular TLR22 acts as an inflammation equalizer via suppression of NF-κB and selective activation of MAPK pathway in fish. Fish Shellfish Immunol. 2018;72:646–57. 10.1016/j.fsi.2017.11.042. 29175443 10.1016/j.fsi.2017.11.042

[237] Gonçalves AC, Pinto AR, Cima A, Olo-Fontinha E, Martins JCL, Garcia J, et al. Plant-derived bioactive compounds: one health perspective. Appl Sci. 2026;16:327. 10.3390/app16010327.

[238] Atanasov AG, Zotchev SB, Dirsch VM, Supuran CT. Natural products in drug discovery: advances and opportunities. Nat Rev Drug Discov. 2021;20:200–16. 10.1038/s41573-020-00114-z. 33510482 10.1038/s41573-020-00114-z PMC7841765

[239] Riley WW, Nickerson JG, Mogg TJ, Burton GW. Oxidized β-carotene is a novel phytochemical immune modulator that supports animal health and performance for antibiotic-free production. Animals. 2023;13. 10.3390/ani13020289. 10.3390/ani13020289 PMC9854599 36670829

[240] Lillehoj H, Liu Y, Calsamiglia S, Fernandez-Miyakawa ME, Chi F, Cravens RL, et al. Phytochemicals as antibiotic alternatives to promote growth and enhance host health. Vet Res. 2018;49:76. 10.1186/s13567-018-0562-6. 30060764 10.1186/s13567-018-0562-6 PMC6066919

[241] Hart CE, Gadiya Y, Kind T, Krettler CA, Gaetz M, Misra BB, et al. Defining the limits of plant chemical space: challenges and estimations. GigaScience. 2025;14:giaf033. 10.1093/gigascience/giaf033. 40184432 10.1093/gigascience/giaf033 PMC11970369

[242] Adetunji AO, Price J, Owusu H, Adewale EF, Adesina PA, Saliu TP, et al. Mechanisms by which phytogenic extracts enhance livestock reproductive health: current insights and future directions. Front Vet Sci. 2025;12. 10.3389/fvets.2025.1568577. 10.3389/fvets.2025.1568577 PMC12042781 40308693

[243] Saha M, Bandyopadhyay PK. In vivo and in vitro antimicrobial activity of phytol, a diterpene molecule, isolated and characterized from Adhatoda vasica Nees. (Acanthaceae), to control severe bacterial disease of ornamental fish, Carassius auratus, caused by Bacillus licheniformis PKBMS16. Microb Pathog. 2020;141:103977. 10.1016/j.micpath.2020.103977. 31953226 10.1016/j.micpath.2020.103977

[244] Reyes-Becerril M, Martínez-Preciado A, Guluarte C, Guerra K, Tovar-Ramirez D, Macias ME, et al. Phytochemical composition and immunobiological activity of Hawthorn Crataegus mexicana nanoencapsulated in Longfin yellowtail Seriola rivoliana leukocytes. Fish Shellfish Immunol. 2019;92:308–14. 10.1016/j.fsi.2019.06.024. 31200073 10.1016/j.fsi.2019.06.024

[245] Reyes-Becerril M, Maldonado-García M, López MG, Calvo-Gómez O, Díaz SM. Cyrtocarpa edulis fruit and its immunostimulant effect on Almaco Jack Seriola rivoliana: in vitro, in vivo and ex vivo studies. Vet Res Commun. 2024;48:1393–407. 10.1007/s11259-024-10309-z. 38285242 10.1007/s11259-024-10309-z

[246] Velázquez-Carriles CA, Angulo C, Macías-Rodríguez ME, Reyes-Becerril M. Phytochemical properties of Cyrtocarpa edulis peel exert antimicrobial activity and enhance immunobiological parameters in Almaco jack Seriola rivoliana cells. Fish Shellfish Immunol. 2025;156:110044. 10.1016/j.fsi.2024.110044. 39608733 10.1016/j.fsi.2024.110044

[247] Disbanchong P, Punmanee W, Srithanasuwan A, Pangprasit N, Wongsawan K, Suriyasathaporn W, et al. Immunomodulatory effects of herbal compounds quercetin and curcumin on cellular and molecular functions of bovine-milk-isolated neutrophils toward Streptococcus agalactiae infection. Animals. 2021;11:3286. 10.3390/ani11113286. 34828017 10.3390/ani11113286 PMC8614355

[248] Suebsaard P, Charerntantanakul W. Rutin, α-tocopherol, and l-ascorbic acid up-regulate type I interferon-regulated gene and type I and II interferon expressions and reduce inflammatory cytokine expressions in monocyte-derived macrophages infected with highly pathogenic porcine reproductive and respiratory syndrome virus. Vet Immunol Immunopathol. 2021;235:110231. 10.1016/j.vetimm.2021.110231. 33740613 10.1016/j.vetimm.2021.110231

[249] Sachan S, Dhama K, Latheef SK, Samad HA, Mariappan AK, Munuswamy P, et al. Immunomodulatory potential of Tinospora cordifolia and CpG ODN (TLR21 agonist) against the very virulent, infectious bursal disease virus in SPF chicks. Vaccines. 2019;7:106. 10.3390/vaccines7030106. 31487960 10.3390/vaccines7030106 PMC6789546

[250] Wu M, Peng M, Zang J, Han S, Li P, Guo S, et al. Phloretin supplementation ameliorates intestinal injury of broilers with necrotic enteritis by alleviating inflammation, enhancing antioxidant capacity, regulating intestinal microbiota, and producing plant secondary metabolites. Poult Sci. 2025;104:105187. 10.1016/j.psj.2025.105187. 40367711 10.1016/j.psj.2025.105187 PMC12142525

[251] Paradowska M, Dunislawska A, Siwek M, Slawinska A. Avian cell culture models to study immunomodulatory properties of bioactive products. Animals. 2022;12. 10.3390/ani12050670. 10.3390/ani12050670 PMC8909239 35268238

[252] Dai L, Yang L, Nedosekov V, Ma J, Fang W, Feng H, et al. Advancements in nanomaterial-based adjuvants for animal vaccines. Mater Today Bio. 2026;38:103123. 10.1016/j.mtbio.2026.103123. 42028278 10.1016/j.mtbio.2026.103123 PMC13101784

[253] Mitchell MJ, Billingsley MM, Haley RM, Wechsler ME, Peppas NA, Langer R. Engineering precision nanoparticles for drug delivery. Nat Rev Drug Discov. 2021;20:101–24. 10.1038/s41573-020-0090-8. 33277608 10.1038/s41573-020-0090-8 PMC7717100

[254] Kane GI, Naylor TE, Lusi EF, Brassil ML, Wigglesworth K, Dinnell RW, et al. Super-adjuvant nanoparticles for platform cancer vaccination. Cell Rep Med. 2025;6:102415. 10.1016/j.xcrm.2025.102415. 41072409 10.1016/j.xcrm.2025.102415 PMC12629812

[255] Calderón-Colón X, Zhang Y, Tiburzi O, Wang J, Hou S, Raimondi G, et al. Design and characterization of lipid nanocarriers for oral delivery of immunotherapeutic peptides. J Biomed Mater Res A. 2023;111:938–49. 10.1002/jbm.a.37477. 36585800 10.1002/jbm.a.37477

[256] Chiang C-Y, Hsieh M-S, Chen M-Y, Tsai Y-W, Lin C-L, Hsu C-W, et al. Lipid nanoparticle-formulated DNA acts as a potent immune modulator for cancer immunotherapy through interferon signaling pathways. Theranostics. 2026;16:2342–56. 10.7150/thno.121364. 41424839 10.7150/thno.121364 PMC12712898

[257] Altammar KA. A review on nanoparticles: characteristics, synthesis, applications, and challenges. Front Microbiol. 2023;14:1155622. 10.3389/fmicb.2023.1155622. 37180257 10.3389/fmicb.2023.1155622 PMC10168541

[258] Kazemi M. Revolutionizing Veterinary Medicine: the role of nanoparticles in advancing animal health, nutrition and disease management. Vet Med Sci. 2025;11:e70528. 10.1002/vms3.70528. 40747873 10.1002/vms3.70528 PMC12314797

[259] Youssef FS, El-Banna HA, Elzorba HY, Galal AM. Application of some nanoparticles in the field of veterinary medicine. Int J Vet Sci Med. 2019;7:78–93. 10.1080/23144599.2019.1691379. 32010725 10.1080/23144599.2019.1691379 PMC6968591

[260] González-Lozano M, Cano-Buendía JA. Chitosan nanoparticles as next-generation carriers for veterinary DNA vaccines: Mechanisms, immune responses, and translational prospects. Vet World. 2025;18:3826–38. 10.14202/vetworld.2025.3826-3838. 41716171 10.14202/vetworld.2025.3826-3838 PMC12913849

[261] Zhou Y, Guo L, Dai G, Li B, Bai Y, Wang W, et al. An overview of polymeric nanoplatforms to deliver veterinary antimicrobials. Nanomaterials. 2024;14:341. 10.3390/nano14040341. 38392714 10.3390/nano14040341 PMC10893358

[262] Zomorodian K, Veisi H, Mousavi SM, Ataabadi MS, Yazdanpanah S, Bagheri J, et al. Modified magnetic nanoparticles by PEG-400-immobilized Ag nanoparticles (Fe3O4@PEG–Ag) as a core/shell nanocomposite and evaluation of its antimicrobial activity. Int J Nanomed. 2018;13:3965–73. 10.2147/IJN.S161002. 10.2147/IJN.S161002 PMC6042532 30022820

[263] Fei X, Jia M, Du X, Yang Y, Zhang R, Shao Z, et al. Green synthesis of silk fibroin-silver nanoparticle composites with effective antibacterial and biofilm-disrupting properties. Biomacromolecules. 2013;14:4483–8. 10.1021/bm4014149. 24171643 10.1021/bm4014149

[264] Guo Y, Awais MM, Fei S, Xia J, Sun J, Feng M. Applications and potentials of a silk fibroin nanoparticle delivery system in animal husbandry. Animals. 2024;14:655. 10.3390/ani14040655. 38396623 10.3390/ani14040655 PMC10885876

[265] Yadav P, Yadav AB, Gaur P, Mishra V, Huma Z-I, Sharma N, et al. Bioengineered Ciprofloxacin-Loaded Chitosan Nanoparticles for the Treatment of Bovine Mastitis. Biomedicines. 2022;10:3282. 10.3390/biomedicines10123282. 36552038 10.3390/biomedicines10123282 PMC9775900

[266] Dizaj SM, Lotfipour F, Barzegar-Jalali M, Zarrintan MH, Adibkia K. Antimicrobial activity of the metals and metal oxide nanoparticles. Mater Sci Eng C Mater Biol Appl. 2014;44:278–84. 10.1016/j.msec.2014.08.031. 25280707 10.1016/j.msec.2014.08.031

[267] El-Maddawy ZK, El-sawy AEF, Ashoura NR, Aboelenin SM, Soliman MM, Ellakany HF, et al. Use of zinc oxide nanoparticles as anticoccidial agents in broiler chickens along with its impact on growth performance, antioxidant status and hematobiochemical profile. Life. 2022;12:74. 10.3390/life12010074. 35054467 10.3390/life12010074 PMC8779200

[268] Kumar N, Kumar S, Singh AK, Gite A, Patole PB, Thorat ST. Exploring mitigating role of zinc nanoparticles on arsenic, ammonia and temperature stress using molecular signature in fish. J Trace Elem Med Biol Organ Soc Min Trace Elem. 2022;74:127076. 10.1016/j.jtemb.2022.127076. 10.1016/j.jtemb.2022.127076 36126543

[269] Bataineh SMB, Arafa IM, Abu-Zreg SM, Al-Gharaibeh MM, Hammouri HM, Tarazi YH, et al. Synergistic effect of magnetic iron oxide nanoparticles with medicinal plant extracts against resistant bacterial strains. Magnetochemistry. 2024;10:49. 10.3390/magnetochemistry10070049.

[270] Reddy PRK, Yasaswini D, Reddy PPR, Kumar DS, Elghandour MMMY, Salem AZM. Nanotechnology in veterinary sector. Handbook of Green and Sustainable Nanotechnology. Cham: Springer. 2023:1541–67. 10.1007/978-3-031-16101-8_8.

